# Recombinant Newcastle disease viruses expressing immunological checkpoint inhibitors induce a pro-inflammatory state and enhance tumor-specific immune responses in two murine models of cancer

**DOI:** 10.3389/fmicb.2024.1325558

**Published:** 2024-01-24

**Authors:** Lisa A. Santry, Jacob P. van Vloten, Amanda W. K. AuYeung, Robert C. Mould, Jacob G. E. Yates, Thomas M. McAusland, James J. Petrik, Pierre P. Major, Byram W. Bridle, Sarah K. Wootton

**Affiliations:** ^1^Department of Pathobiology, Ontario Veterinary College, University of Guelph, Guelph, ON, Canada; ^2^Department of Biomedical Sciences, Ontario Veterinary College, University of Guelph, Guelph, ON, Canada; ^3^Juravinski Cancer Center, Hamilton, ON, Canada

**Keywords:** Newcastle disease virus (NDV), oncolytic virus, immunological checkpoint inhibitors, anti-CTLA-4, PD-1, PD-L1, tumor microenvironment, B16-F10

## Abstract

**Introduction:**

Tumor microenvironments are immunosuppressive due to progressive accumulation of mutations in cancer cells that can drive expression of a range of inhibitory ligands and cytokines, and recruitment of immunomodulatory cells, including myeloid-derived suppressor cells (MDSC), tumor-associated macrophages, and regulatory T cells (Tregs).

**Methods:**

To reverse this immunosuppression, we engineered mesogenic Newcastle disease virus (NDV) to express immunological checkpoint inhibitors anti-cytotoxic T lymphocyte antigen-4 and soluble programmed death protein-1.

**Results:**

Intratumoral administration of recombinant NDV (rNDV) to mice bearing intradermal B16-F10 melanomas or subcutaneous CT26LacZ colon carcinomas led to significant changes in the tumor-infiltrating lymphocyte profiles. Vectorizing immunological checkpoint inhibitors in NDV increased activation of intratumoral natural killer cells and cytotoxic T cells and decreased Tregs and MDSCs, suggesting induction of a pro-inflammatory state with greater infiltration of activated CD8+ T cells. These notable changes translated to higher ratios of activated effector/suppressor tumor-infiltrating lymphocytes in both cancer models, which is a promising prognostic marker. Whereas all rNDV-treated groups showed evidence of tumor regression and increased survival in the CT26LacZ and B16-F10, only treatment with NDV expressing immunological checkpoint blockades led to complete responses compared to tumors treated with NDV only.

**Discussion:**

These data demonstrated that NDV expressing immunological checkpoint inhibitors could reverse the immunosuppressive state of tumor microenvironments and enhance tumor-specific T cell responses.

## Introduction

The goal of cancer immunotherapies is to promote the activation and migration of high-avidity tumor-specific T cells into tumor microenvironments (TMEs) where they can detect and destroy malignant cells (Waldman et al., 2020). However, cancer cells exploit several physiological pathways that limit anti-tumor immune responses, which can reduce the efficacy of immunotherapies (O'Donnell et al., 2019). This includes inhibiting cytotoxic T lymphocytes (CTLs) and effector T cells through immunological checkpoint signaling and enlisting suppressive leukocytes such as CD4 + FoxP3 + regulatory T cells (Tregs). Consequently, a tolerogenic state is established, curtailing T-cell activity against tumor-associated antigens (Hanahan and Weinberg, 2011; Vasievich and Huang, 2011; Pardoll, 2015). To achieve potent immunotherapeutic responses, it is essential to counteract the inhibitory signals that impair T cell functions within the TME. This has been accomplished by antibody-mediated blockade of suppressive immunological checkpoints such as cytotoxic T lymphocyte–associated antigen (CTLA)-4 and programmed cell death protein (PD)-1 (e.g., Ipilimumab and Nivolumab, respectively; Waldman et al., 2020). Both of these receptors are found on activated T cells and are required for maintenance of peripheral self-tolerance and for protection of tissues from damage during immune responses to pathogens (Zappasodi et al., 2018). While CTLA-4 is upregulated early after T cell activation, PD-1 is found on T cells later in the immune response, primarily in peripheral tissues, to modulate effector immune responses (Zhang et al., 2021). Moreover, many cancer types have evolved to express programmed cell death ligand-1 (PD-L1)/PD-L2, the cognate ligands of PD-1, leading to the shut-off of T cell activation in TMEs (Furuse et al., 2020; Rotman et al., 2020; Yamaguchi et al., 2022; Yang et al., 2023).

Due to their ability to significantly improve patient survival, therapeutic monoclonal antibodies targeting CTLA-4 and PD-1/PD-L1 have been approved by the United States Food and Drug Administration (FDA) for the treatment of a range of cancers (Fellner, 2012; Schadendorf et al., 2015; Vallianou et al., 2023). While these immunological checkpoint inhibitors (ICIs) induce a notable increase in CD8^+^ T cell/Treg ratios in tumors, which correlates with increased overall survival in endometrial, ovarian, bladder, and hepatocellular carcinomas (Sato et al., 2005; Cai et al., 2009; de Jong et al., 2009; Leffers et al., 2009), systemic distribution of checkpoint inhibitors can lead to over-activation of a broad array of CTLs, including those that are not specific for cancer cells, leading to severe adverse events such as autoimmunity toward liver, skin, adrenal glands, and the gastrointestinal tract, ultimately necessitating cessation of treatment (Fellner, 2012; Spain et al., 2016; Fessas et al., 2020).

One strategy to avoid the toxicities linked to systemic ICI administration is to modify oncolytic viruses (OVs) to express ICIs (Dias et al., 2012; Kleinpeter et al., 2016; Vijayakumar et al., 2019, 2020). This approach leverages the selective replication of OVs within cancer cells, enabling the localized delivery of ICIs, thereby overcoming some of the challenges of systemic ICI treatment (Kaufman et al., 2015). Additionally, oncolysis of tumor cells by the OV increases pro-inflammatory signaling, tumor-associated antigen release, and recruitment of lymphocytes to the TME (Russell et al., 2019; Burman et al., 2020). This culminates in an anti-tumor immune response, highlighted by the presence of effector natural killer (NK) and T cells. Although it also causes the upregulation of immunological checkpoint molecules, this can be counteracted with by the efficacy of ICIs (Zamarin et al., 2014).

Newcastle disease virus (NDV; recently renamed avian orthoavulavirus-1; Amarasinghe et al., 2018) is an avian paramyxovirus that selectively replicates in tumor cells, inducing both intrinsic and extrinsic apoptosis, as well as necroptosis, leading to the release of cytokines and recruitment of leukocytes to the TME (Elankumaran et al., 2006; Cuadrado-Castano et al., 2015). NDV also promotes substantial increases in immunostimulatory cytokines, as well as activation of CD4^+^ and CD8^+^ T cells, monocytes, and NK cells in the TME (Schirrmacher, 2015; Schirrmacher, 2016). NDV’s tumor-selective replication, oncolytic capacity, and immunostimulatory properties make it a promising cancer therapeutic (Fournier and Schirrmacher, 2013).

In this study, we employed a mesogenic strain of NDV, which is a more fusiogenic version of NDV that unlike lentogenic strains of NDV possess a multi-basic cleavage site within the fusion protein (F) of the virus, known as NDV F3aa. This cleavage site alteration leads to trypsin-independent activation of F, resulting in enhanced replication across multiple cell types, including tumor cells (Buijs et al., 2015) Numerous research investigations have consistently demonstrated that NDV F3aa exhibits superior oncolytic efficacy in murine models compared to lentogenic NDV when administered intratumorally, in contrast to the lentogenic NDV (Silberhumer et al., 2010; Li et al., 2011; Matuszewska et al., 2019; de Graaf et al., 2022). Here we engineered mesogenic NDV, which has a strong safety profile and demonstrated efficacy in clinical trials (Schirrmacher, 2015, 2016) to preferentially express anti-CTLA-4 or soluble PD-1 as checkpoint blockades within TMEs. The impact of NDV-vectored expression of ICIs on tumor-associated immune responses was investigated, revealing that locoregional administration of these recombinant NDVs (rNDV) led to a beneficial shift in leukocyte profiles within tumors, with a notable decrease in suppressive MDSCs and Tregs in TMEs, and a concomitant increase in pro-inflammatory cells leading to an increased ratio of activated effector T cells to inhibitory cells. The NDV expressing ICIs also induced tumor regression and promoted complete responses in two different murine cancer models. This provides rationale for future studies using immunostimulatory oncolytic viruses encoding ICIs.

## Materials and methods

### Ethics statement

All mouse experiments were performed in compliance with the guidelines set forth by the Canadian Council on Animal Care (CCAC). Animal protocols were approved by the Animal Care Committee of the University of Guelph (AUP# 3827). Six-week-old female C57BL/6 and Balb/c mice were purchased from Charles River Laboratories (Wilmington, MA) and housed in a specific pathogen-free isolation facility at the University of Guelph. Mice were allowed to acclimate for 1 week before experiments were initiated. All cell and vector administrations were performed under isoflurane anesthesia, and all efforts were made to minimize suffering. Animal care technicians who measured tumors and determined endpoints were blinded.

### Cells

DF-1 (CRL-12203) and B16-F10 (CRL-6475) cells were obtained from the American Type Culture Collection and CT26-LacZ cells were kindly provided by Dr. John Bell (Ottawa Hospital Research Institute). Cells were cultured in Dulbecco’s Modified Eagles Medium (DMEM) supplemented with 10% fetal bovine serum (FBS) and 2 mM L-Glutamine (ThermoFisher, Canada) and maintained at 37°C and 5% CO_2_ in a humidified incubator.

### Plasmid and vector construction

The full-length cDNA genome of LaSota NDV [pT7NDVF3aa-enhanced green fluorescent protein (GFP)] and helper plasmids (pTM1-NP, pTM1-P, and pTM1-L) were a kind gift from Dr. Peter Palese (Mount Sinai, NY, United States). A leucine to alanine mutation at position 289 was introduced into the fusion gene by site-directed mutagenesis to generate pT7NDV/F3aa/L289A-GFP (refer to Supplementary Table S1 for a list of all primers). To construct rNDV expressing anti-CTLA-4 (αCTLA-4), a DNA fragment encoding the scFv against murine CTLA-4, a (Gly4Ser)3 linker and the hinge CH2-CH3 region of human IgG1 with an N-terminal HA tag (Russell et al., 2019; a kind gift from Andre Lieber, University of Washington Seattle) was synthesized by Invitrogen (GeneArt™ Strings™, Canada) with a signal peptide from the mouse immunoglobulin κ light chain at the 5′ end. The soluble PD-1 fragment representing the extracellular domain from amino acids 21–168 and containing the authentic signal peptide (based on a murine PD-1 mRNA sequence; GenBank accession number X67914.1), was human-mouse codon optimized and synthesized by Invitrogen (GeneArt™ Strings™). In-Fusion cloning™ was used to insert transgenes into the NDV backbone according to the manufacturer’s instructions (Takara, United States). For In-Fusion cloning™, the 5′ end of the primer included 15 bp of homology with each end of the linearized vector including the SacII site. A Flag epitope tag was added to the carboxy terminus of sPD-1 and was included in the reverse primer.

### Production and purification of rNDV

NDV-GFP, NDV-αCTLA-4, and NDV-sPD-1 were rescued using recombinant Modified Vaccinia Ankara (MVA), expressing T7 RNA polymerase, a kind gift from Dr. Bernard Moss (National Institutes of Health), and grown in specific pathogen-free eggs [obtained from the *Canadian Food Inspection Agency* (*CFIA*), Ottawa Ontario Canada] and tittered as previously described (Santry et al., 2018). For each experiment, *in vivo*-quality virus aliquots were removed from a − 80°C freezer, thawed on ice, and reconstituted to the appropriate volume with phosphate-buffered saline (PBS).

### Immunoblotting

Cells of interest were infected with rNDV in six-well culture dishes at a multiplicity of infection (MOI) of 0.1 in a total volume of 500 μL. One hour after the incubation, the inoculum was aspirated, and the cells were incubated at 37°C in 1.5 mL of complete DMEM (cDMEM). After 24 h, cells were pelleted, suspended in 100 μL of radioimmunoprecipitation assay buffer (RIPA) [50 mM Tris pH7.5, 150 mM NaCl, 1% Triton X-100, 0.1% SDS, 10 mM Ethylenediaminetetraacetic acid (EDTA), 1% sodium deoxycholate containing Na_3_VO_4_ (1 mmol/L), NaF (50 mM), and a cocktail of protease inhibitor cocktail (P8340; Sigma-Aldrich, United States)], incubated on ice for 30 min and subjected to centrifugation at ~17,000 × *g* for 30 min to pellet insoluble debris. Clarified cell lysates were separated by sodium dodecyl sulfate polyacrylamide gel electrophoresis (SDS-PAGE; 12% Tris-glycine gel) and transferred to polyvinylidene difluoride (PVDF) membranes (GE Healthcare, Canada). Membranes were blocked in 5% skim milk-PBST (PBS + 0.1% Tween 20) and primary antibodies anti-hemagglutinin (Cat. No. 4724; Cell Signaling Technology), anti-GFP (Cat. No. SAB4301138-100UL; Millipore Sigma), and anti-DYKDDDDK [Flag] (Cat. No. F7425; Sigma), were incubated at a dilution of 1: 1,000 in 1% bovine serum albumin (BSA)-PBST overnight at 4°C. Proteins were detected using horseradish peroxidase (HRP)-conjugated secondary antibodies (Invitrogen) at a 1:2,000 dilution and Western Lightning Chemi-luminescence substrate (Perkin-Elmer, Canada). Images were captured using a ChemiDoc MP Imagine system (BioRad, Canada) and Image Lab software (BioRad, Canada).

### Cell viability assays

Cells were seeded in 96-well plates (5 × 10^4^ cells/well) and allowed to adhere overnight. The following day, wells were inoculated in triplicate with oncolytic rNDVs at MOIs ranging from 0 to 50 plaque-forming units (PFU)/cell. At 48 h post-infection (h.p.i.), resazurin (resazurin sodium salt; Sigma Aldrich) was added to a final concentration of 10 μg/mL. After a 4-h incubation at 37°C in 5% CO_2_, the absorbance was read at a wavelength of 573 nm. For each assay, three experimental replicates with three technical replicates per experiment were conducted. Percent live cells were determined by normalization to mock-infected controls.

### Growth kinetics

All rNDVs were injected into 9-day-old embryonated chicken eggs at 500 PFU in 100 μL. At 50 h.p.i., eggs were moved to 4°C for a minimum of 2 h, to euthanize any live embryos and prevent degradation of dead ones. Allantoic fluid was harvested, and 50% tissue culture infective dose (TCID_50_) assays were performed using DF-1 cells.

### Syncytia formation

Cells were seeded at 5 × 10^5^ cells per well in a six-well plate and allowed to adhere overnight. The next day, cells were infected with NDV-GFP, NDV-αCLTA-4, or NDV-sPD-1 at an MOI of 0.5. Brightfield images of cells were captured with an inverted microscope 24 h.p.i. (Carl Zeiss Axio 154 Observer A1).

### Enzyme-linked immunosorbent assays to detect aCTLA-4 and sPD-1

αCTLA-4 in serum and tissue homogenate samples was detected using a commercial Human IgG ELISA kit (Abcam, ab195215). To detect sPD-1, a 96-well microplate was coated with 100 μg/well of recombinant mouse PD-L1 (RP-88254, ThermoFisher) in PBS (pH 7.4) overnight at 4°C. After washing with TBS-Tween (50 mmol/L Tris–HCl containing 150 mmol/L NaCl and 0.05% Tween 20), the microplate was treated with blocking buffer (TBS-Tween containing 3% calf serum) for 1 h at 37°C, then serum and tissue homogenate samples diluted 1:10 were added to the wells and incubated for 2 h at room temperature. Bound sPD-1 was detected using a rabbit antibody against FLAG (1:2,000, Sigma) and horseradish peroxidase-conjugated goat-anti-rabbit (1: 4,000). After washing, TMB (3,3′,5,5′-tetramethylbenzidine) ELISA Substrate (ThermoFisher) solution was added to each well for developing the color and the absorbance (450 nm) was measured with a Glowmax microplate reader.

### *In vivo* tumor models for leukocyte analysis

B16-F10 melanoma cells (2.5 × 10^5^) in a volume of 30 μL or CT26LacZ colon carcinoma cells (5 × 10^5^) in a volume of 200 μL, suspended in PBS, were injected into the dermis of 6-week-old syngeneic C57BL/6 mice or subcutaneously [proximal to the right inguinal lymph node (LN)] into 6 week-old Balb/c mice, respectively. When tumors reached 5 mm × 5 mm, mice were injected intratumorally with 5 × 10^7^ PFU of NDV-GFP, NDV-αCLTA-4, or NDV-sPD-1 in a total volume of 30 μL, every other day for a total of three injections. Control mice received PBS. Tumors were harvested 36 h after the final injection.

### Leukocyte preparation

Blood was collected from the periorbital sinus of anesthetised mice into heparinized tubes to prevent coagulation. Tumors and tumor-draining inguinal LNs were extracted from euthanized animals. Tumors were weighed, minced, and processed using a gentleMACS dissociator (Miltenyi Biotec; Germany). Following dissociation, cells were filtered through a 70 μm strainer and washed with Hank’s balanced salt solution (HBSS) (ThermoFisher Scientific, Canada). LNs were pressed between the frosted ends of glass microscope slides to make single-cell suspensions, and filtered through a 70 μm strainer. All tissues were treated with an ammonium-chloride-potassium (ACK) buffer (150 mM NH_4_Cl, 10 mM KHCO_3_, 0.1 mM Na_2_EDTA, pH 7.2–7.4), to osmotically lyse red blood cells, and then washed twice with HBSS.

### Surface marker and intracellular staining

Mononuclear cells from blood, LNs, and tumors were treated with anti-CD45.2, a pan leukocyte marker, a viability dye, and antibodies specific to surface markers conjugated to fluorochromes (Refer to Supplementary Figure S1 for a list of flow cytometry reagents and composition of flow panels). For identification of Tregs, cells were permeabilized and fixed with Foxp3 Transcription Factor Staining Buffer Set, which allows the simultaneous analysis of cell surface molecules and intracellular antigens, including nuclear antigens (00-5523-00; ThermoFisher Scientific; Waltham, MA, United States) and stained for Foxp3. Data were acquired using a FACS Canto II flow cytometer with FACSDiva 8.0.1 software (BD Pharmingen; San Jose, CA, United States) and analyzed with FlowJo version 10.2 software (FlowJo LLC, Ashland, OR, United States). For representative dot plots demonstrating the gating strategies used, see Supplementary Figures S2, S3.

### Tumor-specific T cell analysis

Ten days after the first administration of rNDV or PBS, blood-derived leukocytes were collected and prepared as described above. CT26LacZ cells were seeded at a density of 2.5 × 10^5^ in a round-bottom 96-well plate and 16 h later were treated with 50 units of murine interferon (IFN)-γ (mIFN-γ, IF005, Sigma, Canada) for 24 h to maximize the expression of major histocompatibility complex (MHC) class I and/or MHC class II on cancer cells to facilitate effective antigen presentation. Blood-derived leukocytes were co-cultured with or without mIFN-γ-pre-treated CT26LacZ cells for 1 h at 37°C. Next, 1 μg/mL of brefeldin A (ThermoFisher Scientific; Waltham, MA, United States) was added to the cells for 4 h. Surface marker and intracellular staining, as described above, was followed for anti-CD3, anti-CD4, anti-CD8, anti-IFNγ, and anti-TNF-α (Supplementary Figure S1). Data from co-cultured cells had background (from non-co-cultured cells) subtracted to obtain changes in cytokine expression.

### Survival study

CT26LacZ cells (5 × 10^5^) or B16-F10 cells (2.5 × 10^5^) were injected subcutaneously or intradermally between the scapulae of 6-week-old Balb/c or C57BL/6 mice, respectively. When tumors reached approximately 5 mm × 5 mm, mice were injected intratumorally with 5 × 10^7^ PFU of NDV-GFP, NDV-αCLTA-4, or NDV-sPD-1 diluted to a final volume of 40 μL, every other day for 3 days. Control mice received three injections of PBS. Tumor volumes were measured daily for 4 weeks, then every 3–4 days thereafter. Tumor volume was calculated as (length × width^2^)/2, where length was the smaller of the two dimensions measured. Mice were euthanized when tumors reached 20 mm in any direction.

### Statistical analyses

GraphPad Prism version 7 (GraphPad software, San Diego, CA, United States) was used for all graphing and statistical analyses. Differences in leukocyte numbers, ratios, as well as percent and total number of cells expressing functional markers were analyzed by two- or one-way ANOVA with Tukey’s multiple comparisons. Survival curves were estimated by the Kaplan–Meier method, and differences between groups were investigated using the log-rank test. All reported *p* values were two-sided and considered significant if ≤0.05; ^*^p ≤ 0.05, ^**^p ≤ 0.01, ^***^p ≤ 0.001, ^****^p ≤ 0.0001. All graphs show means and standard errors.

## Results

### B16-F10 and CT26LacZ tumor cells upregulated PD-L1 in response to NDV infection

**Figure 1:**
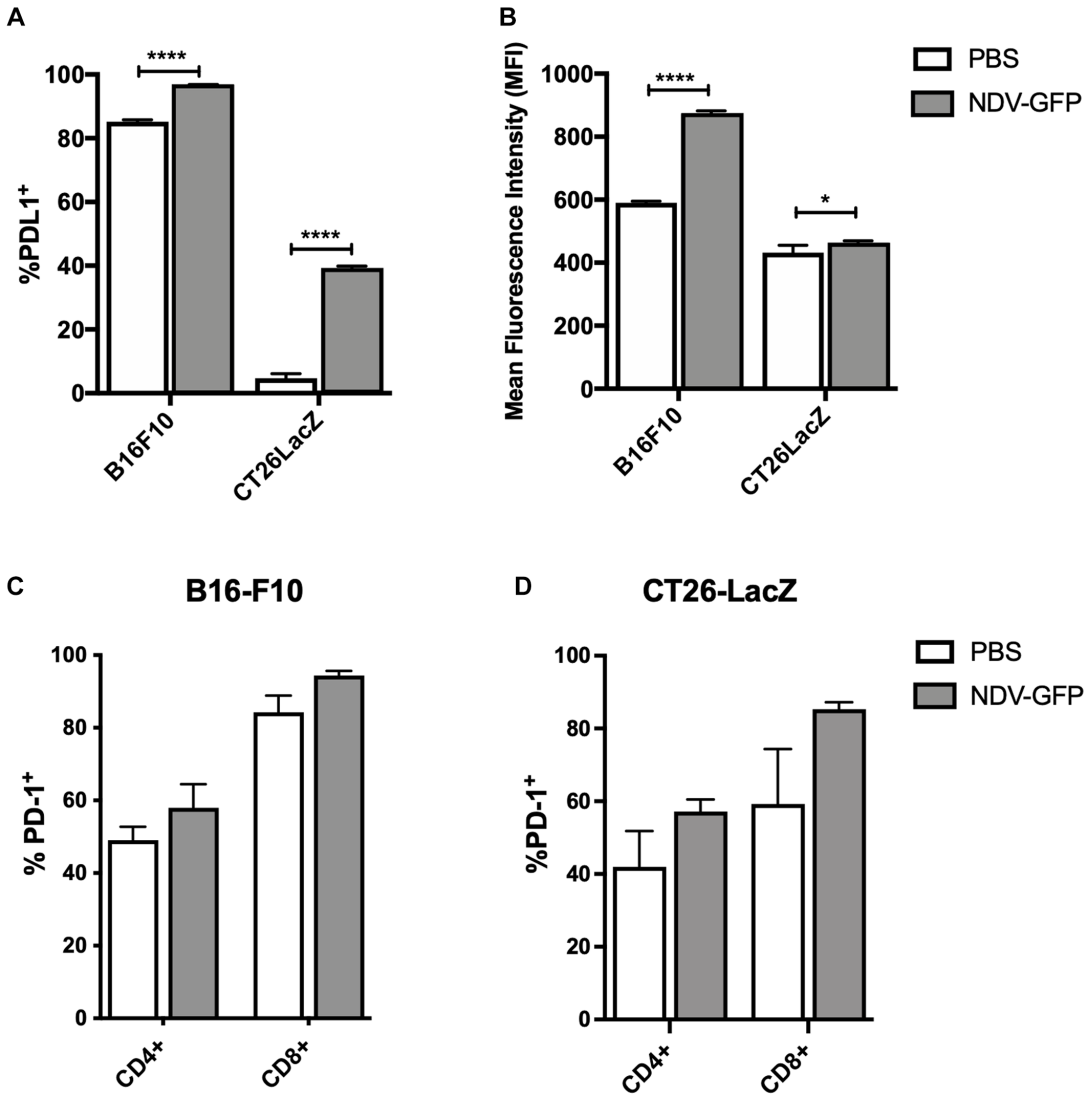
*In vitro* infection of cancer cells with oncolytic Newcastle disease virus (NDV) potentiates surface expression of programmed cell death ligand-1 (PD-L1) and *in vivo* treatment of tumors with NDV promotes expression of programmed cell death protein-1 (PD-1) on CD4+ and CD8+ T-cells. **(A)** Murine B16-F10 melanoma or CT26LacZ colon carcinoma cell lines were treated with NDV encoding green fluorescent protein (GFP) at a multiplicity of infection (MOI) of five for 6 h before quantification of %PD-L1 positive tumor cells **(A)** and mean fluorescence intensity of PD-L1 expression **(B)** by flow cytometry. Once intradermal B16-F10 **(C)** or subcutaneous CT26 LacZ **(D)** tumors (challenge doses of 2.5 × 10^5^ B16-F10 or 5 × 10^5^ or CT26 LacZ cells, respectively) reached ~5 mm × 5 mm, 5 × 10^7^ plaque-forming units (PFU) of NDV-GFP was administered every other day for a total of three doses. Thirty-six hours after the third dose, tumor-infiltrating CD4+ and CD8+ T-cells were analyzed by flow cytometry for expression of PD-1.

To determine whether B16-F10 murine melanoma and CT26LacZ murine colon carcinoma cells expressed and/or upregulated PD-L1 in response to NDV infection, cells were exposed to NDV-GFP at a MOI of five for 6 h, after which PD-L1 expression was analyzed by flow cytometry. Analysis revealed that 85% of B16-F10 cells constitutively expressed PD-L1 and this was further increased to 96% following infection with NDV (*p* < 0.0001; Figure 1A), with a mean fluorescent intensity (MFI) increase of 48% (*p* < 0.0001; Figure 1B). Only 4.7% of CT26LacZ cells expressed PD-L1, but this significantly increased to 39% (*p* < 0.0001) following NDV infection with a modest MFI increase of 7.5% (*p* = 0.0379; Figures 1A,B). To determine how NDV affected PD-1 expression on tumor-resident T cells, 2.5 × 10^5^ B16-F10 cells and 5 × 10^5^ CT26LacZ cells were implanted intradermally and subcutaneously, respectively, and when tumors reached 5 mm × 5 mm, 5 × 10^7^ PFU of NDV-GFP was intratumorally injected every other day for a total of three injections. Tumors were harvested 36 h after the last injection and CD4+ and CD8+ T cells were analyzed for PD-1 expression by flow cytometry. In both B16-F10 (Figure 1C) and CT26LacZ (Figure 1D) tumors, 49 and 42% CD4+ resident T cells expressed PD-1, respectively, which increased to 58% in both tumor types following treatment with NDV. A greater proportion of CD8+ tumor-infiltrating T cells expressed PD-1, at 60% in CT26LacZ tumors and 84.3% in B16-F10 tumors, which increased to 85 and 94.3%, respectively, after NDV treatment. These results provided a rationale for combining NDV with PD-1 blockade.

### Oncolytic NDV could be engineered to express checkpoint inhibitors αCTLA-4, scFV-Fc, and sPD-1

**Figure 2:**
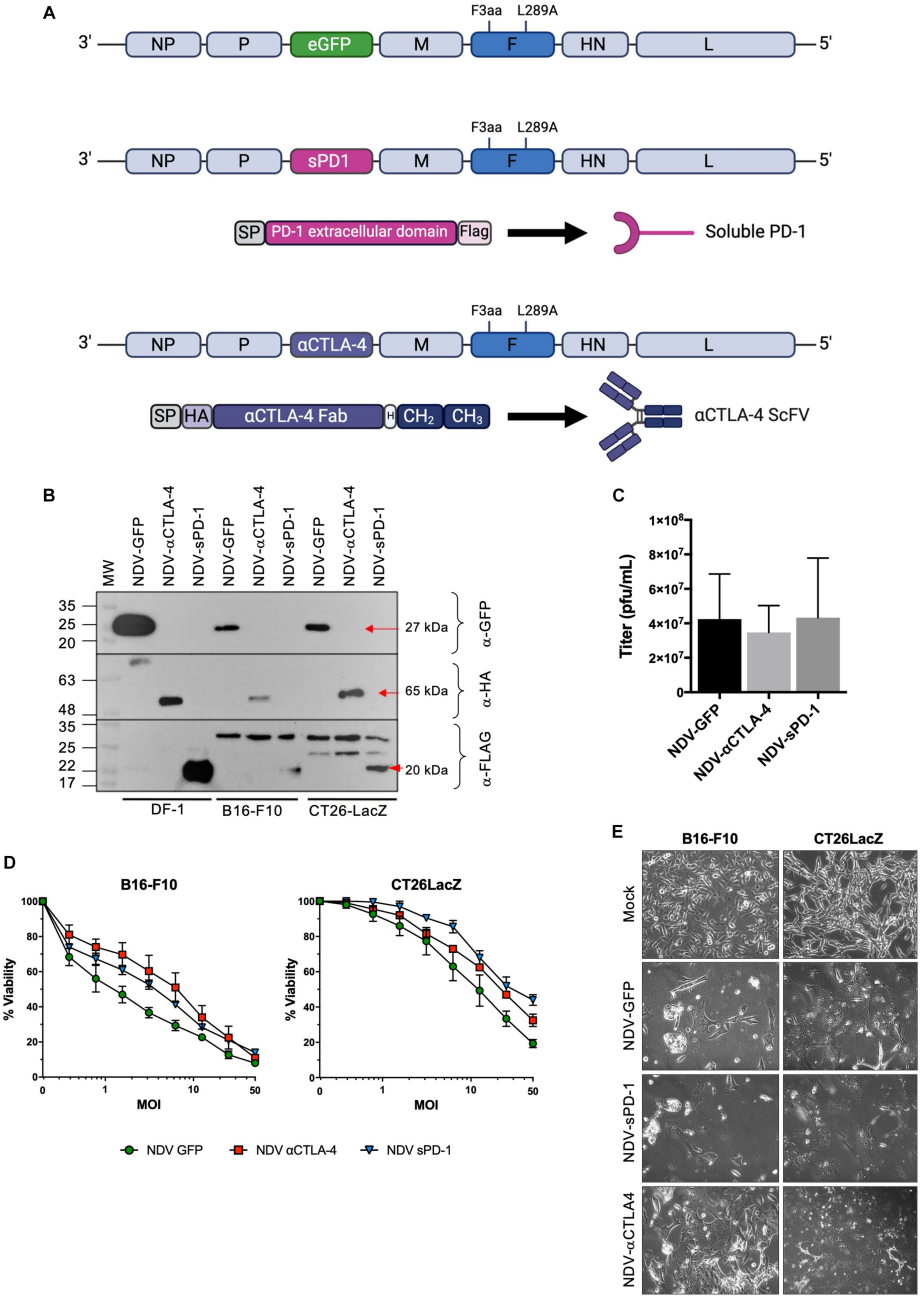
Characterization of recombinant NDV (rNDV) expressing αCTLA-4 and sPD-1. **(A)** Schematic representation of the NDV genome showing the location of the F3aa polybasic cleavage site (nucleotides 5,512–5,526) and L289A (nucleotides 6,043–6,045) modifications in the fusion (F) protein. Using the Sac II site located between the P and M genes, GFP was removed from the plasmid and either an αCTLA4-scFv-Fc or a soluble programmed cell death receptor (sPD-1) transgene was inserted in its place. The αCTLA-4 transgene is comprised of a murine Ig secretion signal peptide (SP), an HA tag, the αCTLA-4 scFv, a hinge (H), and the CH2 and CH3 domains of human IgG1. The sPD-1 construct codes for a murine Ig SP, the extracellular domain of the PD-1 receptor, and a Flag tag on the carboxy terminus. **(B)** Western blot analysis of transgenes expressed from rNDV in various cell lines. Top panel: anti-GFP, middle panel: anti-HA and bottom panel: anti-Flag antibodies were used to detect GFP, αCTLA-4 and sPD-1, respectively, when expressed by NDV. M: molecular markers. Red arrows: indicating the expected molecular weight bands. **(C)** Endpoint titers as determined by TCID_50_ for each rNDV when 500 PFU was inoculated into 9-day-old embryonated chicken eggs and harvested 50 h later. *n* = 5-7, mean + SEM, no significant differences. **(D)** Viability assays performed using rezasurin dye assessing metabolic activity to evaluate changes in oncolytic potential of various NDVs containing transgenes at a range of MOIs. **(E)** Bright-field images of B16-F10 and CT26LacZ cells mock, NDV-GFP, NDV-aCTLA-4, or NDV-sPD-1 infected at an MOI of 0.1 and imaged 24 h post-infection at 20x magnification.

In this study, murine αCTLA-4 scFv fused to the hinge-CH2-CH3 domain of human IgG1 (Tuve et al., 2007) or the extracellular domain of murine PD-1 (sPD-1) comprised of amino acids 21–168 were cloned between the phosphoprotein (P) and matrix (M) genes of NDV(F3aa/L289A), which possesses a multi-basic cleavage site and an L289A mutation in the fusion (F) protein for increased fusogenicity (Vigil et al., 2007; Altomonte et al., 2010), to create NDV(F3aa/L289a)-αCTLA-4 and NDV(F3aa)/(L289a)-sPD-1, respectively (Figure 2A), henceforth referred to as NDV-αCTLA-4 and NDV-sPD-1 for simplicity. Flag and HA epitope tags were added to the carboxy terminus of sPD-1 and amino terminus of αCTLA-4, respectively, to facilitate detection of these proteins. As shown in Figure 2B, western blot analysis of NDV-αCTLA-4 and NDV-sPD-1 infection of B16-F10 and CT26LacZ tumor cells led to robust transgene expression. The avian derived DF1 cell line served as a positive control, as NDV replicates well in these cells. Growth kinetics of the engineered rNDVs were compared to that of NDV-GFP by inoculating nine-day-old embryonated chicken eggs. The allantoic fluid was harvested 50 h post-inoculation and titered by TCID_50_. No significant differences in endpoint titers were observed, suggesting that the NDV-αCTLA-4 and NDV-sPD-1 had similar growth kinetics to NDV-GFP (Figure 2C). Next, B16-F10 and CT26LacZ cell lines were infected with NDV-GFP, NDV-αCLTA-4 and NDV-sPD-1 at increasing MOIs ranging from 0 to 50. Oncolysis was assessed using a resazurin dye-based cell assay, which measures cell metabolism as a proxy for viability, and no significant differences in oncolytic activity were detected (Figure 2D). As expected, differences in susceptibility to rNDV-mediated oncolysis between the B16-F10 and CT26LacZ cell lines were observed, likely due to differing defects in anti-viral and apoptotic signaling pathways (Supplementary Figures S4, S5; Hanahan and Weinberg, 2011; Lemos de Matos et al., 2020) Finally, large multinucleated cells were observed in all cell lines tested, with each virus maintaining its ability to form syncytia with comparable cytopathic effects (Figure 2E). Taken together, these data demonstrated that NDV could be engineered to express ICIs, and this did not compromise the replicative or oncolytic properties of the OV.

### NDV-vectorized immunological checkpoint inhibitors could be detected in the serum but distribution to other tissues was limited to the injected tumor and tumor-draining lymph nodes

To assess the ability of rNDV to serve as both an oncolytic agent and as a vector for localized delivery of ICIs, the distribution of the secreted transgenes in B16-F10 melanoma and CT26LacZ colon carcinoma tumor-bearing mice was evaluated. To establish the models, tumor cells were injected intradermally and subcutaneously, respectively, into the flank proximal to the left inguinal lymph node and allowed to reach 8 mm × 8 mm. NDV-αCTLA-4 and NDV-sPD-1 (5 × 10^7^ PFU) were then injected intratumorally and tissues, including the tumor, heart, liver, both kidneys, tumor-draining inguinal lymph node, axillary lymph node, and serum were collected at 12- and 36-h after administration of NDV. αCTLA-4 and sPD-1 in serum and tissue homogenates were detected using a commercial quantitative human IgG ELISA (Supplementary Figure S6A) and an indirect ELISA (Supplementary Figure S6B), respectively. Intratumoral administration of NDV-αCTLA-4 resulted in expression in three of four the B16-F10 tumors after 12 h and was no longer detected at 36 h. When NDV-αCTLA-4 was administered to CT26LacZ tumors, αCTLA-4 was detected in two of the four tumors after 12 h, and one of the four tumors after 36 h. αCTLA-4 was also detectable in the tumor-draining lymph nodes of the same two mice at 12 h, but not 36 h. While serum αCTLA-4 could be detected in three of four mice after 36 h, it was not detected in any of the other tissues evaluated, including the distal lymph node, liver, spleen, or kidneys. Intratumoral administration of NDV-sPD1 resulted in expression in two of the four B16-F10 tumors after 12 h and all tumors sampled at 36 h. Similarly, sPD-1 was detected in two of the four CT26LacZ tumors after 12 h and three of the four tumors sampled at 36 h. While sPD-1 was detected in the serum of all but one B16-F10 tumor-bearing mouse at both 12- and 36-h, as well as all CT26-LacZ tumor-bearing mice regardless of timepoint, sPD-1 was not detected in any other tissue examined. Of note, we did not observe any evidence of toxicity as evidenced by weight loss, or other outward clinical signs such as lethargy, hunched posture, squinty eyes, etc.

### Intratumoral administration of rNDV expressing immunological checkpoint inhibitors altered tumor-infiltrating lymphocyte and other immune cell profiles toward a pro-inflammatory state in the B16-F10 murine melanoma model

**Figure 3:**
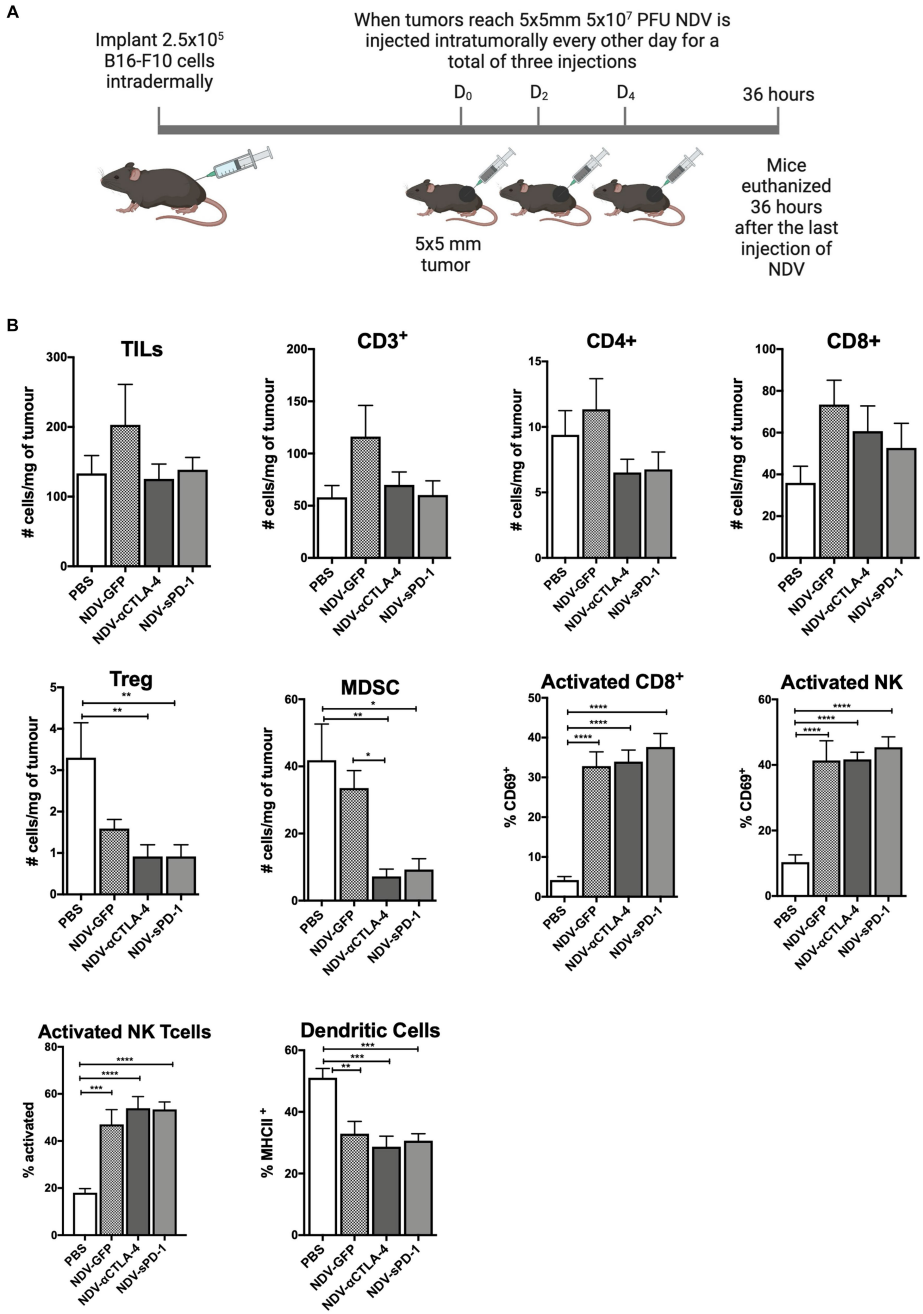
Intratumoral administration of recombinant NDV (rNDV) into B16-F10 tumors shifts the TIL other immune cell profiles toward a pro-inflammatory state. **(A)** 2.5 × 10^5^ B16-F10 cells were injected intradermally into 6-week-old C57BL/6 mice. Once tumors reached 5 mm × 5 mm, 5 × 10^7^ PFU of rNDV expressing GFP, αCTLA-4, or sPD-1 was injected intratumorally every other day for a total of three injections. Thirty-six hours after the last injection, whole blood, lymph nodes, and tumors were harvested and prepared for flow cytometric analysis. **(B)** Absolute numbers or frequencies of various immunological subsets from B16-F10 tumors were identified via flow cytometry and expressed as number of cells per g of tumor. All graphs show means + SE. Data represents results from two independent experiments with *n* = 6-8 per group. ^*^p < 0.05, ^**^p < 0.01, ^***^p < 0.001, and ^****^p < 0.0001. TILs, Tumor-infiltrating lymphocytes; MDSC, Myeloid derived suppressor cells; and Treg, T regulatory cells.

Recombinant NDV therapies were first evaluated in the syngeneic and orthotopic B16-F10 murine melanoma model in C57BL/6 mice to investigate differences in leukocyte infiltration in treated vs. untreated tumors. B16-F10 cells were injected intradermally into C57BL/6 mice. When tumors reached approximately 5 mm × 5 mm, rNDV injections (5 × 10^7^ PFU per injection) were initiated and administered every other day for a total of three injections. Thirty-six hours after the last injection, tumors were collected for analysis of leukocyte profiles (Figure 3A). While others have reported an increase in the number of leukocytes infiltrating the TME after treatment with NDV (Zamarin et al., 2009, 2014, 2017), the goal was to provide a more comprehensive analysis of the leukocyte profile, including evaluation of the activation status of various leukocytes. Therefore, in addition to evaluating CD45.2^+^ leukocytes, NK cells and CD8^+^ and CD4^+^ T lymphocytes, NKT cells, B lymphocytes, neutrophils, MDSCs, and dendritic cells (DC) were included in the leukocyte panel (see Supplementary Figure S1 for leukocyte markers used). Furthermore, the assessment included activation markers for CD4^+^, CD8^+^, NK and NKT cells, as well as MHC class II as a maturation marker for DCs.

When analyzing TILs and other immune cells from mice treated with different rNDVs, there were no significant differences in the overall number of TILs, including those expressing CD3^+^, CD4^+^, and/or CD8^+^ (Figure 3B). However, treatment with NDV-αCTLA-4 or NDV-sPD-1 led to a decrease in the number of intratumoural Foxp3^+^ Tregs (*p* < 0.01).In addition, there was a decrease in the number of MDSCs in tumors from mice treated with NDV-αCTLA-4 (*p* < 0.01) and NDV-sPD-1 (*p* < 0.001) compared to PBS-treated and NDV-GFP-infected controls (*p* < 0.05 and *p* < 0.01, respectively). While there were no changes in the absolute numbers of CD8^+^ cells, there was a statistically significant increase in expression of the early activation marker, CD69, on CD8^+^ cells from mice treated with NDV-GFP (*p* > 0.001), as well as both NDV-αCTLA-4 and NDV-sPD-1 (*p* < 0.0001), when compared to PBS-treated controls. There was a significant increase in the percentage of activated NK cells (CD69^+^) in mice treated with NDV-GFP (*p* < 0.01), NDV-αCTLA-4 (*p* < 0.001), and NDV-sPD-1 (*p* < 0.001) as well as an increase in activated NKT (CD69^+^) cells in mice treated with NDV-GFP (*p* < 0.001), NDV-αCTLA-4 (*p* < 0.0001), and NDV-sPD-1 (*p* < 0.0001) when compared to PBS-treated controls, as indicated by increased expression of the early activation marker CD69. Finally, there were no differences in the number of macrophages, neutrophils, or B cells (data not shown). However, there was a decrease in the percent of mature DC’s in B16-F10 tumor-bearing mice treated with any of the rNDVs.

### Intratumoral administration of rNDV expressing immunological checkpoint inhibitors had a modest effect on TIL and other immune cell profiles in CT26-LacZ tumor-bearing mice

**Figure 4:**
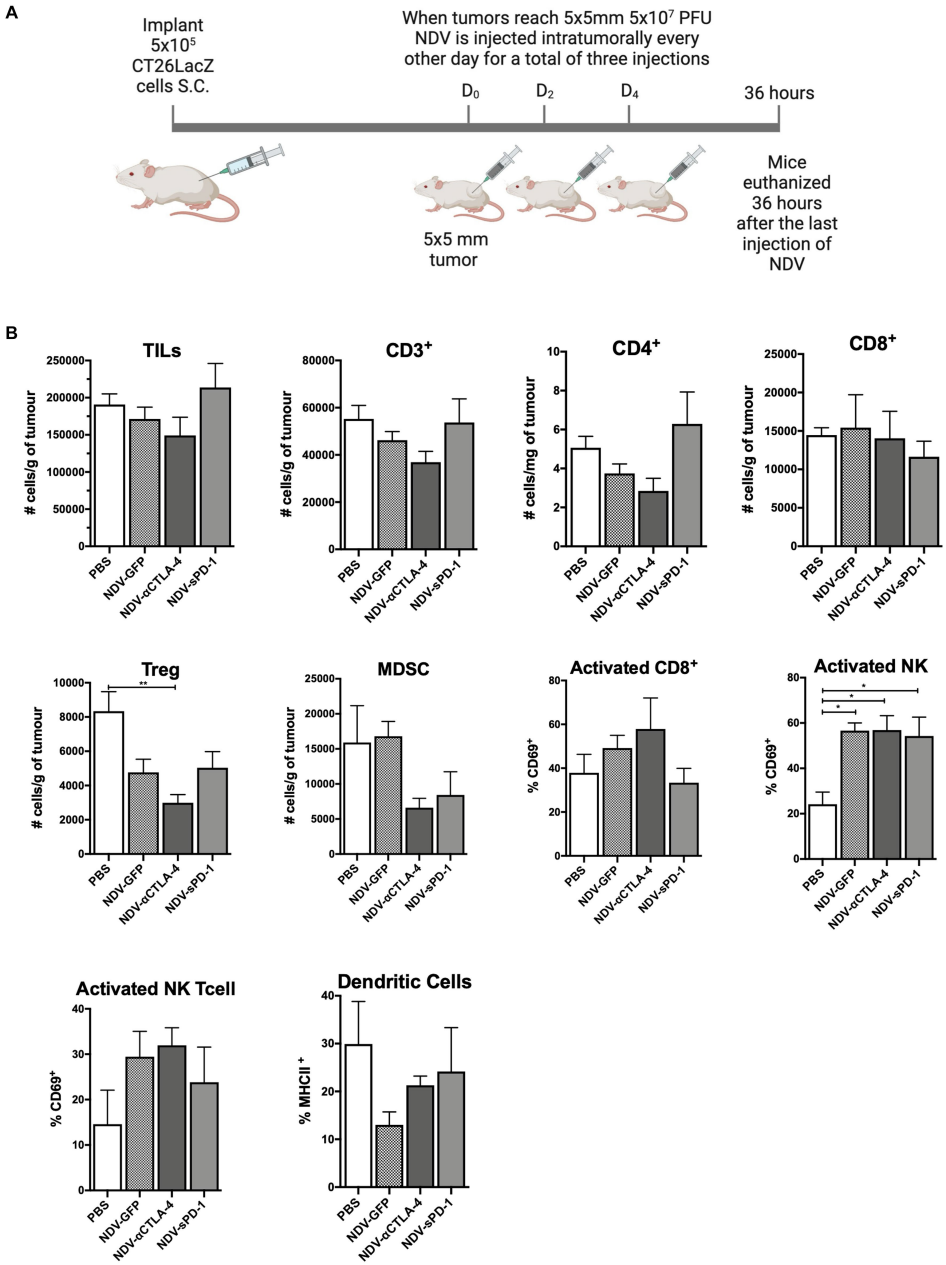
Intratumoral administration of recombinant NDV (rNDV) into CT26LacZ tumors shifts the TIL other immune cell profiles toward a pro-inflammatory state. **(A)** 5 × 10^5^ CT26LacZ cells were injected subcutaneously into the lower flank of 6-week-old Balb/C mice. Once tumors reached 5 mm × 5 mm, 5 × 10^7^ PFU of rNDV expressing GFP, αCTLA-4, or sPD-1 was injected intratumorally every other day for a total of three injections. Thirty-six hours after the last injection, whole blood, lymph nodes and tumors were harvested and prepared for flow cytometric analysis. **(B)** Absolute numbers or frequencies of various immunological subsets from CT26LacZ tumors were identified via flow cytometry and expressed as number of cells per g of tumor. All graphs show means + SE. Data represent results from two independent experiments with *n* = 5 per group. ^*^p < 0.05, ^**^p < 0.01, ^***^p < 0.001, and ^****^p < 0.0001. TILs, Tumor-infiltrating lymphocytes; MDSC, Myeloid derived suppressor cells; and Treg, T regulatory cells.

To evaluate how rNDVs impacted the immune response in a different tumor model and a different strain of mice, 5 × 10^5^ CT26LacZ cells were injected subcutaneously into the flanks of Balb/c mice. Following a similar schedule, once tumors reached 5 mm × 5 mm, rNDV injections (5 × 10^7^ PFU per injection) were initiated and administered every other day for a total of three injections. Thirty-six hours after the last injection tumors were collected for analysis of leukocyte profiles (Figure 4A). In the CT26LacZ tumors, treatment with rNDVs had no significant effect on the overall number of TILs compared to PBS-treated tumors. However, treatment with NDV-αCLTA-4 led to a reduction in the number of Tregs in tumors (*p* < 0.01) compared to PBS-treated tumors (Figure 4B), and for all NDV treatments, there was a significant increase (*p* < 0.05) in the number of activated NK cells in tumors as indicated by elevated expression of CD69. Overall, the difference in TIL and other immune cell profiles and their concomitant cellular activation was less pronounced in the CT26LacZ model compared to the B16F-10 model.

### Treatment with rNDV expressing immunological checkpoint inhibitors changed pro-inflammatory/suppressive cell ratios in both B16-F10 and CT26LacZ cancer models

**Figure 5:**
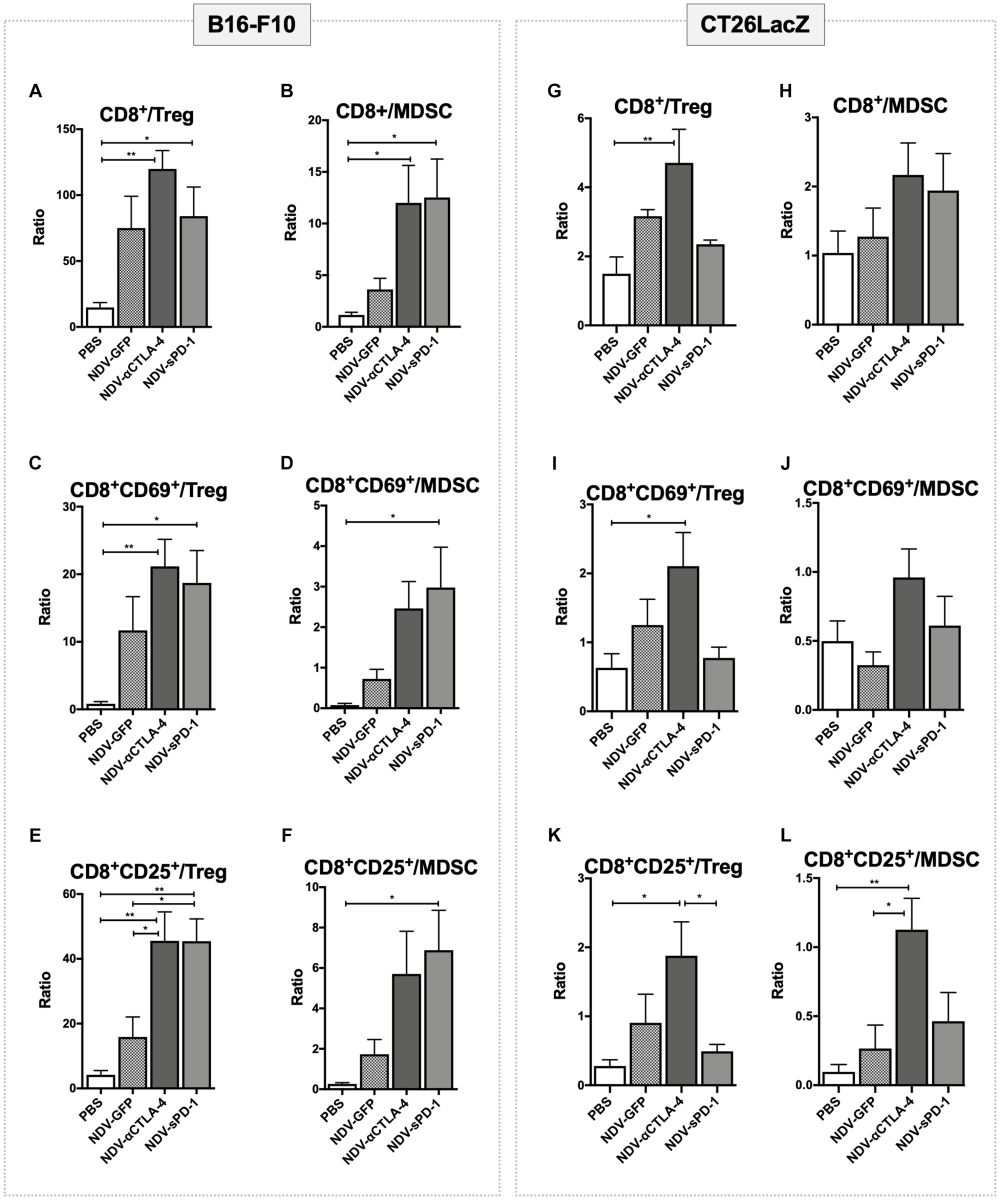
Treatment with recombinant NDV (rNDV) leads to changes in pro-inflammatory/suppressive cell ratios in both B16-F10 and CT26LacZ cancer models. 2.5 x 10^5^ B16-F10 cells (left panel) were implanted intradermally or 5 x 10^5^ CT26LacZ cells (right panel) were injected subcutaneously into C57BL/6 or Balb/c mice, respectively. Once tumors reached 5 x 5mm they were injected with PBS or 5 x 10^7^ PFU of NDV-GFP, NDV-αCTLA-4, or NDV-sPD-1 every other day for a total of three injections. Tumors were harvested 36 hours following the last NDV injection and the ratios of various immunological subsets (indicated above the graphs) identified from tumors via flow cytometry were evaluated. All graphs show means + SE. *n* =5 per group. *P < 0.05, **P < 0.01, ***P < 0.001, ****P < 0.0001.

Despite the overall reduction in the number of suppressive cells in tumors treated with NDV-αCTLA-4, and in some cases NDV-sPD-1 (Figures 3B, 4B), whether NDV induced a pro-inflammatory state was unclear. To address this question, pro-inflammatory/suppressive cell ratios from tumors described in Figures 3, 4 were further analyzed. Analysis of leukocyte ratios in B16-F10 tumors revealed significant increases in CD8^+^/Treg ratios for mice treated with NDV encoding αCTLA-4 or sPD-1 transgenes, compared to PBS-treated controls (*p* < 0.01 and *p* < 0.05, respectively; Figure 5A) as well as increased ratios for CD8^+^/MDSC (*p* < 0.05 for both; Figure 5B). Interestingly, evaluation of early (CD69) and late (CD25) activation markers revealed a promising shift in activated leukocyte ratios in mice treated with NDV expressing ICI transgenes. With respect to early activated CD8^+^ cells, NDV-αCTLA-4 and NDV-sPD-1 caused greater shifts in the CD8^+^CD69^+^/Treg ratios (*p* < 0.01 and *p* < 0.05, respectively; Figure 5C) and CD8^+^CD69^+^/MDSC ratios (Figure 5D) compared to PBS-treated mice (*p* < 0.01 and *p* < 0.05, respectively). Remarkably, evaluation of late activation markers demonstrated a substantially improved response with a higher CD8^+^CD25^+^/Treg ratio in NDV-αCTLA-4- and NDV-sPD-1-treated mice compared not only to mock (*p* < 0.01), but also to NDV-GFP (*p* < 0.05) treatment groups (Figure 5E). Only treatment with NDV-sPD-1 showed a significant increase in the CD8^+^CD25^+^/MDSC ratio (Figure 5F).

In the CT26LacZ tumors some statistical differences in the lymphocyte ratios were observed, albeit to a lesser extent than the B16-F10 tumors. Differences were detected in the CD8^+^/Treg ratios of the NDV-αCTLA-4 treatment group compared to PBS-treated controls and the NDV-sPD-1 treatment group (*p* < 0.01, *p* < 0.05, respectively; Figure 5G). Similar results were observed for the CD8^+^CD69^+^/Treg (Figure 5I) and CD8^+^CD25^+^/Treg ratios compared to PBS-treated controls (*p* < 0.05; Figure 5K). It was only the CD8^+^CD25^+^/MDSC ratios where a difference was observed between NDV-αCTLA-4 and NDV-GFP as well as NDV-αCTLA-4 and PBS-treated groups (*p* < 0.05 and *p* < 0.01, respectively; Figure 5L).

### Treatment with rNDV expressing immunological checkpoint inhibitors led to tumor regression that was durable in the B16-F10 mouse model

**Figure 6:**
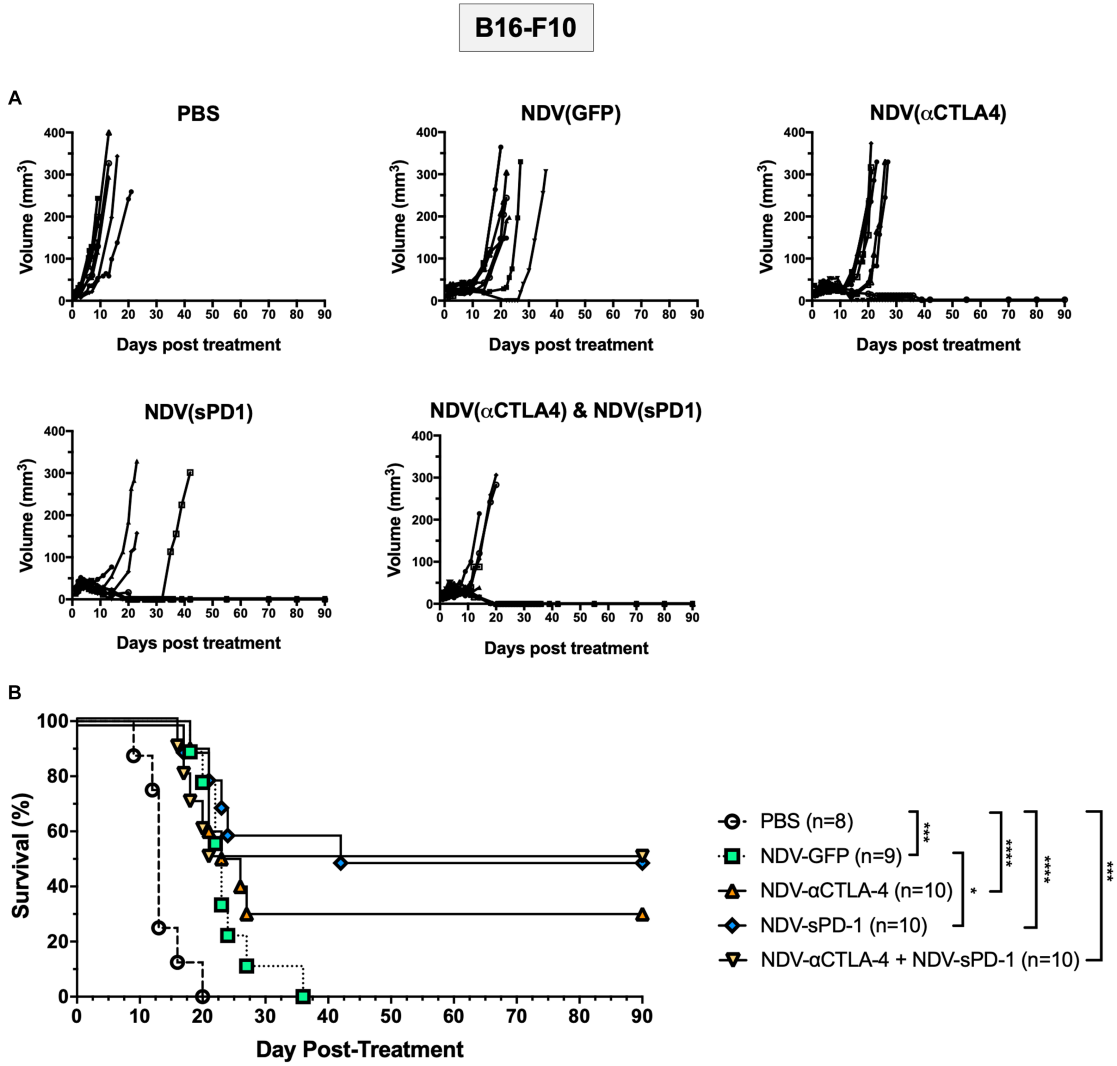
Survival of B16-F10 tumor bearing mice following treatment with recombinant NDVs (rNDVs). 5 × 10^5^ B16-F10 cells were implanted intradermally into C57BL/6 mice (*n* = 8–10). Once tumors had reached 5 mm × 5 mm in size they were inoculated with PBS, 5 × 10^7^ PFU of NDV-GFP, NDV-αCTLA-4, NDV-sPD-1, or both NDV-αCTLA-4 and NDV-sPD-1 every other day for a total of three injections. **(A)** Tumors were measured daily for 4 weeks, then every 3–4 days. **(B)** Survival curve following treatment with PBS, NDV-GFP, NDV-αCTLA-4, NDV-sPD-1,or both NDV-αCTLA-4 and NDV-sPD-1. Mice were euthanized when tumors reached 20 mm in any direction. Significance was estimated using Kaplan–Meier. ^*^p < 0.05, ^**^p < 0.01, ^***^p < 0.001, and ^****^p < 0.0001.

To test the efficacy of rNDVs encoding ICIs, survival studies were conducted in the B16-F10 murine melanoma model. When tumors reached 5 mm × 5 mm, intratumoral injections of 5 × 10^7^ PFU were administered as before, and tumors were measured in two directions every day until mice reached endpoint (defined as 20 mm in diameter in any direction). While intratumoral injections of all rNDVs led to delayed growth compared to PBS-treated mice, there was ensuing outgrowth in the NDV-GFP-treated groups (Figure 6A). However, a subset of mice receiving NDV expressing ICIs experienced complete responses (CR): NDV-αCTLA-4 had 30% CR, while NDV-sPD-1 and NDV-αCTLA-4 + NDV-sPD-1 had 50% CR (Figure 6B). All treatments with rNDV resulted in significant increases in the overall survival, even NDV-GFP, which increased average survival from 13.6 to 23.9 days (*p* < 0.001). However, only treatment with NDV-sPD-1 significantly increased overall survival compared to NDV-GFP treated mice (*p* < 0.05; Figure 6B).

### Treatment with rNDV expressing immunological checkpoint inhibitors leads to tumor regression and increased complete responses in the CT26LacZ mouse model

**Figure 7:**
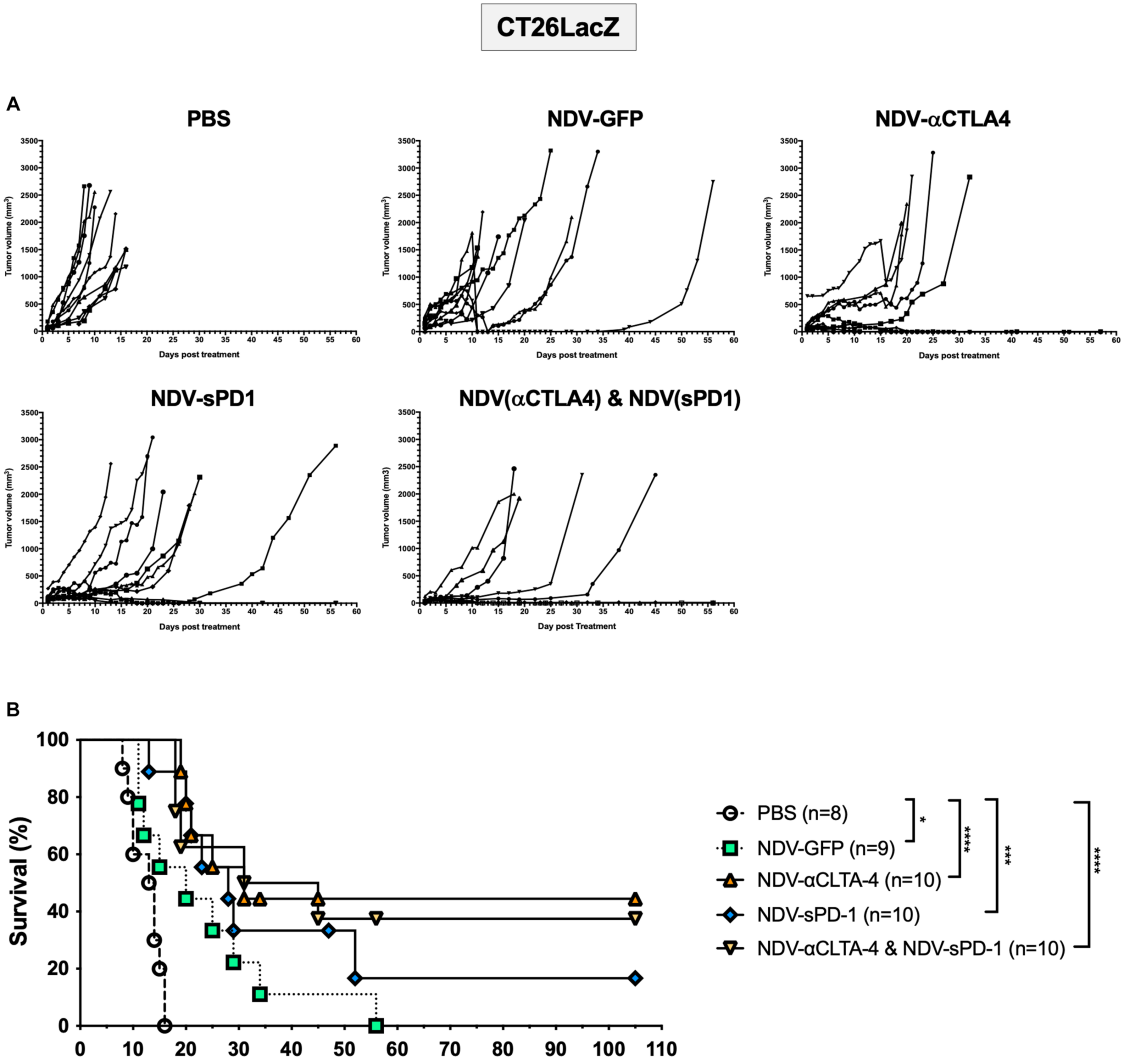
Survival of CT26 LacZ tumor bearing mice following treatment with recombinant NDVs (rNDVs). 5 × 10^5^ CT26 LacZ cells were injected subcutaneously into Balb/c mice (*n* = 8–10). Once tumors had reached 5 mm × 5 mm in size they were inoculated with PBS, 5 × 10^7^ PFU of NDV-GFP, NDV-αCTLA-4, NDV-sPD-1, or both NDV-αCTLA-4 and NDV-sPD-1 every other day for a total of three injections. **(A)** Tumors were measured daily for 4 weeks, then every 3–4 days. **(B)** Survival curve following treatment with PBS, NDV-GFP, NDV-αCTLA-4, NDV-sPD-1, or both NDV-αCTLA-4 and NDV-sPD-1. Mice were euthanized when tumors reached 20 mm in any direction. Significance was estimated using Kaplan–Meier. ^*^p < 0.05, ^**^p < 0.01, ^***^p < 0.001, and ^****^p < 0.0001.

Once again, to compare differences in cancer models and mouse strains, the CT26LacZ tumor model was employed. This model was chosen since it is refractory to anti-viral IFN signaling (Supplementary Figure S5) and was able to support NDV-mediated transgene expression 24 h after three successive injections with NDV-GFP (Supplementary Figure S7B). Balb/c mice were injected subcutaneously with 5 × 10^5^ CT26LacZ cells between the scapulae of 6-week-old mice. When tumors reached 5 mm × 5mm, treatment with PBS or NDV-GFP, NDV-αCTLA-4, or NDV-sPD-1 (5 × 10^7^ PFU) was initiated. After treatment with rNDVs, many of the tumors began to develop scabs. In some instances, these scabs would fall off, as indicated by a large decrease in tumor volume (Figure 7A). However, the tumors grew back in a portion of the rNDV-treated groups and mice eventually reached endpoint. On average, PBS-treated control mice reached endpoint on day 12.5 post-treatment (Figure 7B). Compared to PBS treatment, all NDV-treated groups had significantly increased overall survival [NDV-GFP (*p* < 0.05), NDV-αCTLA-4 (*p* < 0.0001), NDV-sPD-1 (*p* < 0.001), and NDV-αCTLA-4 + NDV-sPD-1 (*p* < 0.0001)]. Mice that received intratumoral injections of NDV-αCTLA-4 had the greatest survival benefit with 44.4% CR, while NDV-sPD-1 had only 16.67% CR, and NDV-αCTLA-4 + NDV-sPD-1 had 37.50% CR.

### Treatment with rNDV expressing immunological checkpoint blockades increased the percentage of functional tumor-specific T cells

**Figure 8:**
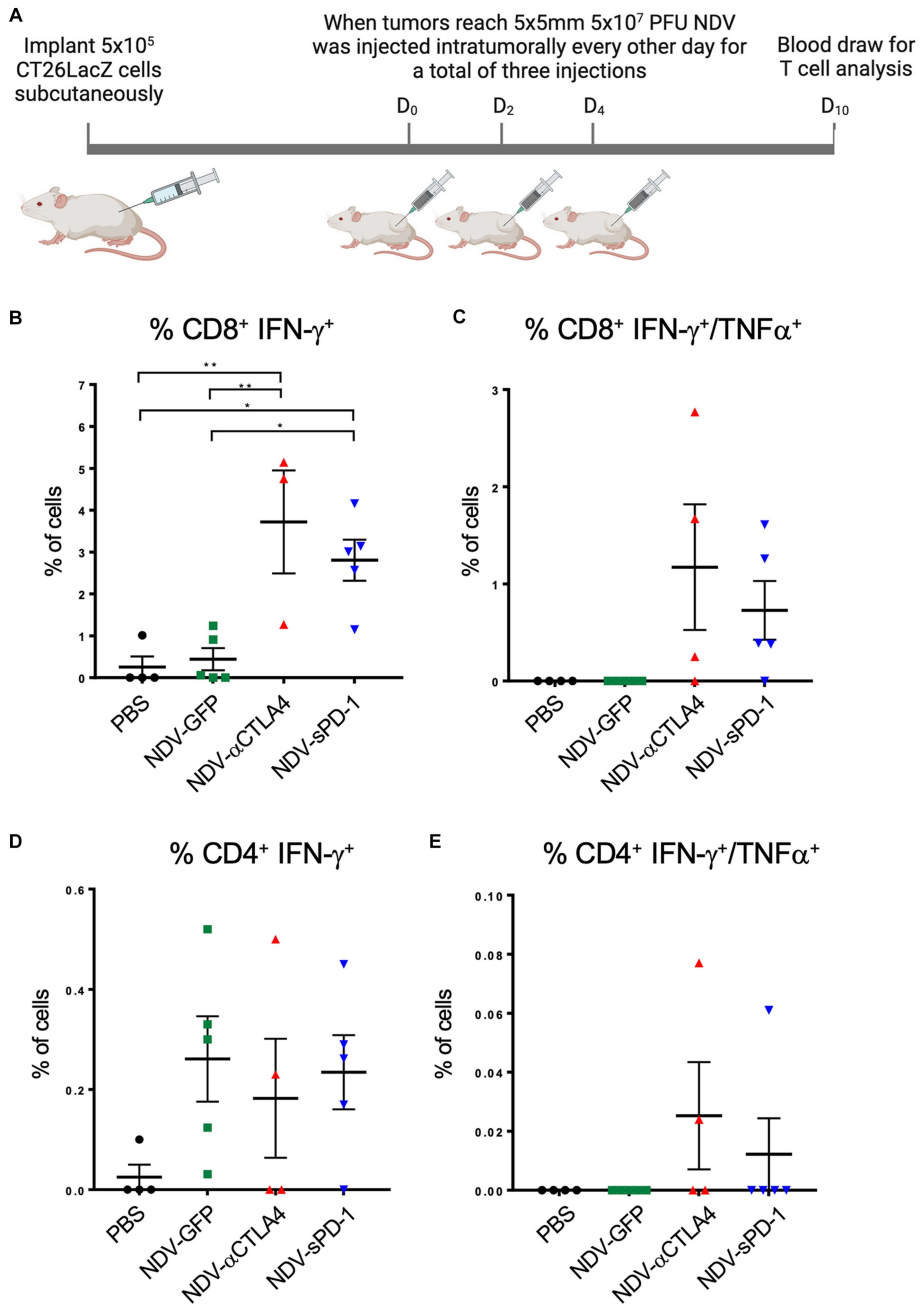
Quantifying CT26 LacZ specific T cell responses following treatment with recombinant NDVs (rNDVs). **(A)** CT26 LacZ tumors (5 x 5mm) were inoculated with PBS or 5 x 10^7^ PFU of NDV-GFP, NDV-αCTLA-4 or NDV-sPD1 every other day for a total of three injections. Ten days following the first injection, blood was collected via retro-orbital vein and co-cultured with IFNγ stimulated or unstimulated CT26 LacZ cells for 1 hours before surface marker and intracellular staining. The percentage of CT26 LacZ tumor specific CD8^+^ IFNγ^+^ **(B)**, CD8^+^ IFNγ^+^ TNF^+^ **(C)**, CD4^+^ IFNγ^+^ **(D)**, CD4^+^ IFNγ^+^ TNF^+^ **(E)** T cells were quantified by flow cytometry. Values are relative to those obtained from non-co-cultured cells. All graphs show means + SE. *P<0.05, **P < 0.01, ***P < 0.001, ****P < 0.0001.

Immunological checkpoint inhibitors anti-CTLA-4 and anti-PD-1 prevent the shutdown of T cells by binding to CTLA-4 on Tregs, thereby decreasing the number of Tregs (Simpson et al., 2013; Romano et al., 2015) and MDSCs (de Coaña et al., 2017), and by blocking PD-L1 and PD-L2 from binding to PD-1 receptors on T cells, respectively. Therefore, it was hypothesized that there might be an increase in the percentage of tumor-specific T cells in mice treated with rNDV expressing αCTLA-4 or sPD-1. To test this hypothesis, 10 days after the first treatment with rNDV, retro-orbital blood samples were taken (Figure 8A) and T cell function was assessed by measuring production of IFN-γ and TNF-α by flow cytometry after re-stimulation with CT26LacZ tumor cell lysates as described previously (van Vloten et al., 2019). In mice treated with NDV-αCTLA-4 or NDV-sPD-1, there was an increase in the number of CT26LacZ tumor-specific CD8+ cells compared to PBS-treated mice (*p* < 0.01 and *p* < 0.05, respectively), as well as compared to NDV-GFP-treated mice (*p* < 0.01 and *p* < 0.05, respectively; Figure 8B). However, no significant differences were observed in CD8+ T cells expressing both type II interferon (IFN-γ) and tumor necrosis factor (TNF-α), even though 3/4 and 4/5 mice from the NDV-αCTLA-4 and NDV-sPD-1-treated groups, respectively, had CD8+ cells expressing both cytokines, while the NDV-GFP- and PBS-treated groups did not (Figure 8C). Evaluation of CD4+ cells did not reveal any significant differences in IFN-γ or TNF-α production (Figures 8D,E).

## Discussion

While published data affirm NDV as a promising oncolytic virotherapy, our goal was to further improve its efficacy by combining the immunostimulatory properties of mesogenic NDV with its potential to concentrate expression of checkpoint inhibitors within the TME, leading to a reversal of immunosuppression and/or tolerization. In the current study, we engineered NDV to express a CTLA-4-specific scFv fused to the hinge-CH2-CH3 domain of human IgG1 to target CTLA-4 receptors on Tregs. We chose to express an scFv-Fc as it is a more compact alternative to the IgG format and retains many of the desirable characteristics of IgG, including bivalency, a relatively long half-life, solubility, antibody-dependent cellular cytotoxicity (ADCC), and complement-dependent cytotoxicity (Bujak et al., 2014). Moreover, human IgG1 functions similarly to murine IgG2a (Nimmerjahn et al., 2005), which is a strong inducer of ADCC and antibody-dependent cellular phagocytosis in murine NK cells, polymorphonuclear leukocytes, as well macrophages (Nimmerjahn and Ravetch, 2008; Overdijk et al., 2012; Dekkers et al., 2017). We also engineered NDV to express a soluble version of the murine PD-1 receptor to block both PD-L1 and PD-L2, which are often expressed on tumor cells and other TILs, from binding PD-1 receptors on CTLs and rendering them dysfunctional. This is of importance since PD-L1 antibodies do not block PD-L2, which has been found to be expressed by tumor cells, especially when PD-L1 is blocked (Overdijk et al., 2012). We selected the soluble PD-1 receptor for expression due to its demonstrated efficacy in cancer treatment (Xiao et al., 2007; Pan et al., 2013; Sorensen et al., 2016), its small size to minimize interference with NDV replication, and to reduce the risk of off-target ADCC. Based on the ELISA data shown in Supplementary Figure S6, it would appear that recombinant NDV expresses larger amounts of sPD-1 *in vivo* than αCTLA-4. This could be due to the smaller size of sPD-1 or the due to the fact that it is a less complex molecule than αCTLA-4, which is expressed as a single polyprotein that requires cleavage by a 2A self-cleaving peptide in order for the heavy and light chain domains of the αCTLA-4 immunoadhesin to assemble and be secreted. While NDV has previously been engineered to express the immunological checkpoint inhibitors, α-CTLA-4, α-PD-1, and α-PD-L1, this was done in the context of a lentogenic (non-fusogenic) strain of NDV, which has somewhat reduced replicative and fusogenic ability due to its requirement for trypsin-like proteases for cleavage and activation of the viral fusion protein (Zamarin and Palese, 2012; Vijayakumar et al., 2020).

Following exposure of tumor cells to NDV *in vitro*, more CT26LacZ cells were stimulated to express PD-L1 but the total amount of PD-L1 expressed on a per cell basis was not dramatically increased. One possible implication for the increase in the number of tumor cells expressing PD-L1 is the increased potential for T cell inhibition in the TME. This might explain why there were significantly more tumor specific CD8+ T cells in the NDV-sPD-1 treated mice compared to NDV-GFP treated mice, as shown in Figure 8B. This would also imply that anti-PD-L1 combination therapy might synergize with oncolytic NDV treatment.

Treatment with rNDV expressing αCTLA-4 or sPD-1 altered the lymphocyte profile in the TME to a pro-inflammatory state through increased ratios of CD8^+^/Treg and CD8^+^/MDSC. As hypothesized, NDV-αCTLA-4 led to an overall decrease in Treg numbers. However, a significant reduction in Treg numbers was also detected in the B16-F10 model after treatment with NDV-sPD-1. While there was a trend toward decreased Treg numbers in NDV-GFP treated tumors, this did not reach statistical significance. It is possible that our sampling time was not ideal and that if we had included a later sampling point, we might have observed a further reduction in Tregs in the mice treated NDV-αCTLA-4. Nevertheless, there are reports demonstrating that blocking PD-1 binding prevents conversion of CD4^+^ T cells to Tregs and suggests that the PD-1 signaling plays a role in conversion of conventional T cells to Tregs (Dyck et al., 2016). While we cannot confirm that this occurred in NDV-sPD-1-treated tumors, our data would support a role for PD-1 signaling in the conversion of CD4^+^ T cells to Tregs. In addition to a decrease in Tregs after treatment with rNDV-αCTLA-4, a reduction in the number of MDSCs was also observed. There are reports of ipilimumab leading to a decline in MDSCs, although the mechanism is not well understood (de Coaña et al., 2017; Pico de Coaña et al., 2020). MDSCs are not known to express CTLA-4 receptors; however, expansion and recruitment of these cell types can occur in a Treg-dependent manner (Holmgaard et al., 2015), suggesting that the rNDV-αCTLA-4-mediated reduction in MDSCs that was observed may have been an indirect effect due in part to a decrease in Treg numbers.

The primary mode of action of anti-CTLA-4 is through ADCC targeting of Tregs (Selby et al., 2013; Simpson et al., 2013; Romano et al., 2015). Ipilimumab has a human IgG1 Fc domain which binds to the hFCγRIIIa (CD16a) receptor on NK cells, nonclassical monocytes (Romano et al., 2015), and macrophages (Simpson et al., 2013), leading to ADCC-dependent lysis of targeted cells. Our data suggest that ADCC may be occurring in our model due to the decrease in overall Treg numbers. However, we also observed a decrease, albeit not significant, in conventional T cells that could potentially decrease efficacy of the treatment, as there have been discrepancies regarding the importance of the role of CD4^+^ versus CD8^+^ cells in tumor clearance (Shedlock and Shen, 2003; Bevan, 2004; Perez-Diez et al., 2007).

Our data also revealed a significant decrease in the number of tumor-associated macrophages (TAMs) after treatment with NDV-αCTLA-4 or NDV-sPD-1 compared to NDV-GFP, which in the latter case, showed a surprisingly significant increase in TAMs. There is currently much debate over the role of macrophages in tumorigenesis. One meta-analysis found that 10/15 studies evaluating TAMs and prognosis found that macrophage density was correlated with poor prognosis, with only 3/15 studies indicating macrophage infiltration was a strong predictor of survival (Bingle et al., 2002). This is likely because TAMs are comprised of M1 and M2 macrophages, both with opposing functions in immunomodulation. M1 macrophages are associated with strong effector functions, whereas M2 macrophages play a role in regulation of immunity, maintenance of tolerance, and tissue repair/wound healing (Italiani and Boraschi, 2014). Further studies characterizing these two subtypes of TAMs would be of great interest, especially since mice treated with NDV-αCTLA or NDV-sPD-1 had a reduction in the overall number of macrophages in the TME in the CT26LacZ model. In fact, Tregs and MDSCs can produce IL-10, IL-4, and IL-13 and induce the suppressive form of macrophages (Taams et al., 2005; Sinha et al., 2007; Tiemessen et al., 2007; DeNardo et al., 2009) and we speculate a drop in these suppressive cells and cytokines would subsequently reduce the M2 macrophage burden, hopefully leading to a better prognosis (Mantovani and Locati, 2013; Zhang et al., 2014; Mills et al., 2017).

While immunological profiling provides insight into mechanisms of action and treatment efficacy, it is the effector T cell to Treg cell ratios favoring a pro-inflammatory environment that are strongly associated with response to therapy and better outcomes in patients (Sato et al., 2005; Biller et al., 2010; Baras et al., 2016). Here we show that rNDV expressing immunological checkpoint blockades are effective in their ability to increase the ratio of CD8^+^ T cells to both Treg and MDSC suppressive cells, especially after treatment with NDV-αCTLA-4, and this was observed in both mouse models. NDV-sPD-1, on the other hand, was much more efficacious in the B16-F10 model. While many studies have reported a large influx of tumor-infiltrating CTLs and NK cells after treatment with NDV, most studies did not characterize the activation status of these TILs. Here, we used CD25 as an activation marker for conventional CD4^+^ T cells (Tconv) and CD69 in combination with CD25 as activation markers for CD8^+^ T cells, rather than grouping all effector cells together without consideration for their activation status. In both tumor models an increase in the percentage of activated CD8^+^ T cells was observed when treating with rNDV, especially NDV-αCLTA-4. When the ratio of effector cells to either Tregs or MDSCs was assessed, the results were promising in the B16-F10 model. However, only when the ratios of activated effector cells to both Treg and MDSCs were evaluated, was there a substantial enhancement in both cancer models, which would strongly suggest steering TMEs toward a pro-inflammatory state.

More promising results were observed in the B16-F10 model with respect to TIL and other immune cell analysis with a greater shift in stimulatory/suppressive cell ratios compared to the CT26LacZ tumor model, which correlated with better transgene expression (Supplementary Figure S7). Further studies are needed to evaluate whether these differences were due to differences in the genetic background of the mice (C57BL/6 mice preferentially develop T helper cell 1-driven immune responses whereas Balb/c mice are a prototypic T helper cell 2-biased strain (Stewart et al., 2002; Watanabe et al., 2004; Schulte et al., 2008; Mills et al., 2017)), the nature of the tumor cell line, or dissimilarities in the environment in which the cells were implanted (subcutaneous versus intradermal).

Treatment with rNDVs led to an increase in the size of tumor-draining LNs (Supplementary Figure S8) and a sizeable increase in the percentage of CD69 and CD25 activated CD8^+^ cytotoxic T cells was detected in inguinal tumor-draining LNs after treatments with any of the three rNDVs (Supplementary Figures S9, S10). However, there was a concomitant increase in LN-resident Tregs and MDSCs in both models, and a corresponding decrease in pro-inflammatory-to-suppressive cell ratios, which warrants concern (Supplementary Figures S9, S10). Although the exact mechanisms contributing to the change in leukocyte populations is not known, LNs are a crucial site for T cell activation. Here, Tregs directly interact in a CTLA-4-dependent manner with activated DCs and conventional T cells to reduce antigen presentation and activation of T cells through release of inhibitory cytokines by Tregs (Thornton and Shevach, 2000; Piccirillo and Shevach, 2001; Pico de Coaña et al., 2014; Matheu et al., 2015). This might explain why we did not see a large increase in the percentage of activated Tconv in the LNs. MDSCs are even less well understood, but are believed to exhibit suppressive functions through cell-to-cell contact and are known to secrete a large array of inhibitory cytokines, as well as arginase, reactive oxygen species (ROS) and inducible nitric oxide synthase (iNOS; reviewed in (Veglia et al., 2021)). Regardless, as mentioned previously, we still observed a sizeable increase in the percentage of early and sustained activated CD8^+^ T cells in the inguinal LNs.

Expression of anti-CTLA-4 and sPD-1 from NDV should concentrate these therapeutic transgenes in the TME, where they will drain to LNs along with OV-released tumor associated antigens (TAAs) to enhance the activation of tumor-specific T cells while avoiding the toxicities associated with traditional systemic delivery (Fellner, 2012; Spain et al., 2016). However, we do not know whether limiting ICI distribution to within the tumor may reduce the induction of systemic immune responses. Our results indicated no drop in the percentage of Tregs in the inguinal LN (Supplementary Figures S8, S9) or in the blood (data not shown), which suggests there is, at most, limited αCTLA-4 leaving the TME. One caveat is that NDV-mediated transgene expression in murine cancer cells is markedly less efficient than in human cancer cells (Zamarin et al., 2017). However, to evaluate immunotherapies in an experimental model, the animals must have an intact immune system, which limits us to using mouse models of cancer. Therefore, the amount of transgene expression from NDV, and hence the therapeutic benefit in mice, is likely and underestimation of what would be observed in human tumors. Indeed, Dmitry et al., found that injections of NDV led to upregulation of CTLA-4 on TILs in contralateral tumors and only NDV in combination with anti-CTLA-4 led to rejection of pre-established distant tumors (Zamarin et al., 2014). Vijayakumar et al. (2020) assessed the impact of various immunostimulatory combinations of lentogenic NDV and only NDV-mediated blockade of PD-L1 provided a survival benefit in a unilateral tumor model, with fewer mice exhibiting a CR than observed in our study, possibly due to our use of mesogenic NDV rather than lentogenic NDV. Interestingly, Vijayakumar et al. (2020) also found that systemic CTLA-4 blockade in addition to treatment with rNDV improved tumor rejection in the bilateral melanoma model. Therefore, it is possible that combining mesogenic NDV with anti-CTLA-4 treatment, which can be administered at higher doses and at different time intervals, may greatly improve systemic antitumor immune responses.

Despite detecting an increase in T effector cells expressing markers of early (CD69) and late (CD25) activation, it was important to confirm that these cells were tumor-specific. Indeed, we were able to demonstrate the existence of functional CD4^+^ and CD8^+^ tumor-specific T cells and found a greater number of these cells when tumors were injected with NDV-αCTLA-4 or NDV-sPD-1 compared to the PBS- and NDV-GFP-treated controls. We observed a large increase in the percentage of CD8^+^ cells expressing IFNγ after treatment with NDV-αCTLA-4 or NDV-sPD-1. However, the effect was more pronounced with NDV-αCTLA-4. These findings provide evidence for an increase in the quality of T cells, and more specifically, activation of tumor-specific T cells, and suggest a decrease in T cell anergy caused by Tregs and MDSCs. Surprisingly, there were no significant differences in the percentage of activated (CD25) Tconvs in the tumor. Some reports suggest a crucial role for Tconv in tumor clearance (Perez-Diez et al., 2007; Kreiter et al., 2015), while others propose that they are dispensable (Zamarin et al., 2017), which might be due to their ability to aide in robust activation of CD8^+^ T cells, macrophages, and NK cells (Shedlock and Shen, 2003; Bevan, 2004; Williams et al., 2006; Livingstone et al., 2009). However, we are unsure to what extent they aided in clearance of cancer cells in our models.

Survival of mice bearing B16-F10 and CT26LacZ tumors was significantly extended when treated with NDVs expressing checkpoint blockade inhibitors as compared to PBS-treated controls. When comparing NDV-sPD-1 and NDV-αCTLA-4, NDV-αCTLA-4 prolonged survival the most in the CT26 LacZ model (44.4 vs. 16.67% CR) while NDV-sPD1 prolonged survival the most in the B16-F10 model (50 vs. 30% CR). This suggests that tumor-specific T cells, limited by tumor cell expression of PD-L1 may exist in greater numbers in B16-F10 tumors, explaining why PD-1 blockade was more successful in B16-F10 and CTLA-4 blockade in CT26LacZ models. Additional intrinsic differences such as tumor mutational burden, which is greater in melanomas, may also have played a role (Sha et al., 2020; Li et al., 2021). Surprisingly, combination of both NDVs and dual blockade of PD-L1 and CTLA-4 did not have a synergistic effect in either of the cancer models we evaluated (Figures 6B, 7B). Given that previous studies have observed complementary activity of PD-L1 and CTLA-4 blockade (Bowyer et al., 2016; Wolchok et al., 2017, 2022), it is possible that the concentration of ICIs or the dosing regimen we used was not optimal for blockade of both PD-L1 and CTLA-4 in these tumor models, thus further studies are required.

In summary, rNDVs were successfully engineered to express molecules that can block immunological checkpoints and provide proof-of-principle that locoregional delivery of these rNDVs leads to a shift in leukocyte profiles within tumors that have been shown to correlate with better prognoses. We demonstrated a notable decrease in suppressive MDSC and Treg cells in the TME and a concomitant increase in pro-inflammatory cells, leading to increased ratios of activated effector T cells to inhibitory cells. The data presented here provide a rationale for future studies using immunostimulatory oncolytic viruses engineered to express immunological checkpoint blockades.

## Data availability statement

The original contributions presented in the study are included in the article/Supplementary material, further inquiries can be directed to the corresponding author.

## Ethics statement

All mouse experiments were performed in compliance with the guidelines set forth by the Canadian Council on Animal Care (CCAC). Animal protocols were approved by the Animal Care Committee of the University of Guelph (AUP# 3827). The study was conducted in accordance with the local legislation and institutional requirements.

## Author contributions

LS: Conceptualization, Data curation, Formal analysis, Investigation, Methodology, Writing – original draft, Writing – review & editing. JV: Formal analysis, Investigation, Methodology, Writing – review & editing. AA: Formal analysis, Methodology, Writing – review & editing. RM: Formal analysis, Investigation, Methodology, Writing – review & editing. JY: Data curation, Writing – review & editing. TM: Data curation, Formal analysis, Methodology, Writing – review & editing. JP: Resources, Supervision, Writing – review & editing. PM: Conceptualization, Funding acquisition, Resources, Writing – review & editing. BB: Conceptualization, Data curation, Formal analysis, Funding acquisition, Investigation, Project administration, Resources, Supervision, Writing – review & editing. SW: Conceptualization, Data curation, Formal analysis, Funding acquisition, Investigation, Methodology, Project administration, Resources, Supervision, Writing – original draft, Writing – review & editing.
