# Supplementary material for: Recombinant Newcastle disease viruses expressing immunological checkpoint inhibitors induce a pro-inflammatory state and enhance tumor-specific immune responses in two murine models of cancer
Source: Front Microbiol. 2024 Jan 24;15:1325558. doi: 10.3389/fmicb.2024.1325558 (PMC10847535; doi:10.3389/fmicb.2024.1325558)
Supplement: Supplementary file 1 [file Data_Sheet_1.PDF]

Name	Sequence 5'→ 3'
**InFusion Fwd**	ACCGAGTTCCCCCCGCGGTTAGAAAAAATACGGGTAGAA**CC GCCACCATG**GAGACAGACACACTCCTGCTATGGG
**hIgG1 InFusion RV**	TTGGACCTTGGGTCCGCGGTTCATCATTTACCCGGAGACAG
**sPD1 InFusion RV**	TTGGACCTTGGGTCCGCGGATTACTTGTCATCGTCGTCT
**L289A-Mutagenesis Fwd**	GGTATACAGGTAACT**GCA**CCTTCAGTCGGGAACC
**L289A-Mutagenesis RV**	GGTTCCCGACTGAAGG**TGC**AGTTACCTGTATACC
**Sequence Fwd**	GTTCTTCAGCCAAGCTCCTAAGC
**Sequence RV**	GGGCAGAATCAAAGTACAGCCC

Table S1. Primers used for Infusion cloning, L289A mutagenesis and sequencing of recombinant Newcastle disease viruses (NDV). The forward (Fwd) infusion primers were designed to contain 15 base pairs (bps) of homology to the NDV(F3aa/L289A) backbone (double underline), followed by a SacII cut site (blue), the regulatory gene end (GE), intergenic (IG) and gene start (GS) sequences (underlined), which are important for regulating the viral polymerase activity between the different open reading frames, a Kozak sequence to improve translation initiation (bold) and a portion of the signal peptide from the mouse immunoglobulin κ light chain in order to bind to the synthesized GeneArt™. The reverse (RV) infusion primers were designed to include a SacII site (blue) at the 5'end as well as extra base pairs downstream of the stop codon so that the insert would adhere to the "rule of six", followed by 15 bps overlap with the NDV backbone. Mutagenesis primers show three nucleotides modified to change the codon from an L to an A (green). Sequencing primers were designed to flank inserts to ensure the correct transgene was incorporated.

Panel	Leukocyte subset (phenotypes)	Antibody	Clone	Company	Catalogue #
**Natural Killer Cells (NK)**	Pan leukocyte marker: CD45.2^{+}	Anti-CD45.2 PE-Cy2	104	BD Pharmingen; San Jose, USA	560696
	NK cells: CD3^{+}, NK1.1^{+}	Anti-CD3-BV421	1 45-2C11	BD Horizon; San Jose, USA	562600
	CD8 T cells: CD3^{+}, CD8^{+}	Anti-CD8-BV510	53-6.7	BD Horizon	563068
	Activation marker: CD69^{+}	Anti-NK1.1 APC	PK136	BD Pharmingen	550627
		Anti-CD69 FITC	H1.2F3	BD Pharmingen	557392
		7-Aminoactinomycin D		ThermoFisher Scientific; Waltham, USA	00-6993-50
**B cell**	Pan leukocyte marker: CD45.2^{+}	Anti-CD45.2 PE-Cy2	104	BD Pharmingen	560696
	B cells: CD3^{-}, CD19^{+}	Anti-CD3-BV421	1 45-2C11	BD Horizon	562600
	Plasma cells: CD3^{-}, CD38^{+}, CD138^{+}	Anti-CD8-BV510	53-6.7	BD Horizon	563068
	CD8 T cells: CD3^{+}, CD8^{+}	Anti-CD19 FITC	1D3	BD Pharmingen	553785
		Anti-CD38 AlexaFluor647	90	BD Pharmingen	562769
		Anti-CD138-PE	281-2	BD Pharmingen	553714
		7-Aminoactinomycin D		ThermoFisher Scientific	00-6993-50
**T cell**	Pan leukocyte marker: CD45.2^{+}	Anti-CD45.2 PE-Cy2	104	BD Horizon	560696
	CD4 T cells: CD3^{+}, CD4^{+}	Anti-CD3-BV421	1 45-2C11	BD Horizon	562600
	Conventional T cells: CD3^{+}, CD4^{+}	Anti-CD8-BV510	53-6.7	ThermoFisher Scientific	563068
	T regulatory cells: CD3^{+}, CD4^{+}	Anti-CD4 FITC	RM4-4	ThermoFisher Scientific	11-0043-85
	CD8 T cells: CD3^{+}, CD8^{+}	Anti-Foxp3 PE	MF23	BD Pharmingen	560414
		Anti-CD25 APC	PC61.5	ThermoFisher Scientific	17-0251-82
	Activation Marker: CD25^{+}	Anti-CD279 (PD-1) PerCP efluor710	J43	ThermoFisher Scientific	46-9985-82
	PD-1 Receptor: CD279^{+}	Fixable viability dye efluor780		ThermoFisher Scientific	65-0865-14
**Myeloid**	Neutrophils: CD11b^{+},CD11c low, Ly6G^{+}	Anti-CD11b eFluor 450	M1/70	ThermoFisher Scientific	48-0112-82
		Anti-CD11c PE-Cy7	N418	ThermoFisher Scientific	25-0114-82
	Eosinophils: CD11b^{+}, F4/80^{+}, Ly6G^{-}, SiglecF^{+}	Anti-Ly6C PAC-Cy7	AL-21	BD Pharmingen	560596
		Ati-Ly6G PE	1A8	BD Pharmingen	551461
	Macrophages:CD11b^{+}, F4/80^{+}, Ly6G^{-}, SiglecF^{-}, Ly6C^{-}	Anti-F4/80 FITC	BM8	ThermoFisher Scientific	11-4201-85
	Dendritic cells: CD11b (low to bright), CD11c^{+}	Anti-CD170 (Siglec F) PerCP efluor710	1RNM44N	ThermoFisher Scientific	46-1702-82
		Anti-MHC II APC	M5/114.15.2	ThermoFisher Scientific	47-5321-82
	Myeloid derived suppressor cells: CD11b^{+},F4/80^{+},SiglecF^{-}, Ly6G^{-}, Ly6C^{+}	eFluor506 Fixable Viability Dye		ThermoFisher Scientific	65-0866-14
**Tumor- specific Tcells**	CD4+ and CD8+	Anti-CD3-BV421	1 45-2C11	BD Horizon; San Jose, USA	562600
		Anti-CD8-BV510	53-6.7	BD Horizon; San Jose, USA	563068
		Anti-CD4-FITC	RM4-4	ThermoFisher Scientific; Waltham, USA	11-0043-85
		Anti-IFNγ-APC	XMG1.2	ThermoFisher Scientific; Waltham, USA	17-7311-82
		Anti-TNFα-PE	MP6-XT22	ThermoFisher Scientific; Waltham, USA	12-7321-82

Figure S1. List of antibodies used in flow cytometry to identify leukocyte subsets.

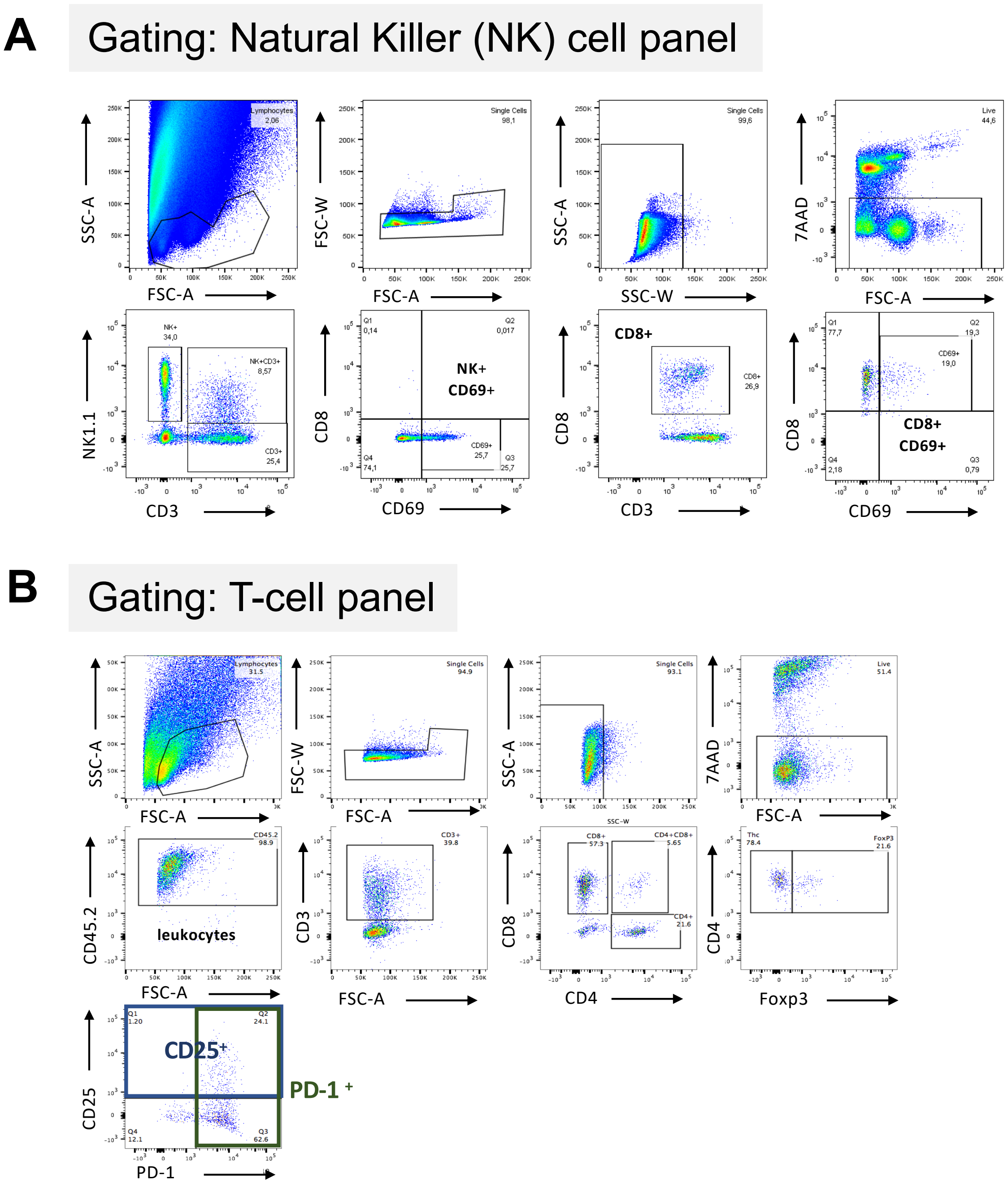

Figure S2. Representative dot plots from CT26LacZ tumours demonstrating the flow cytometry gating strategies used to identify leukocyte subsets. Similar strategies were used for blood-derived mononuclear cells and lymph node gating, as well as other tissues from B16-F10 mice. Leukocytes were first gated based on forward and side-scatter area (FSC-A vs. SSC-A). Doublets were excluded, followed by dead cells, using 7-Aminoactinomycin D (7AAD) staining. Leukocytes were then identified by CD45.2 expression. (A) NK cells were identified by negative CD3 staining and positive NK1.1 staining. CD8 T cells were identified by positive CD3 and CD8 staining. CD69 expression was then assessed for early activation. (B) CD8 T cells were identified by positive CD3 and CD8 staining. CD4 T cells were identified by positive CD3 and CD4 staining. Regulatory and conventional helper T cells were further identified by FoxP3 staining. CD25 expression was then assessed for activation, as well as the PD-1 receptor.

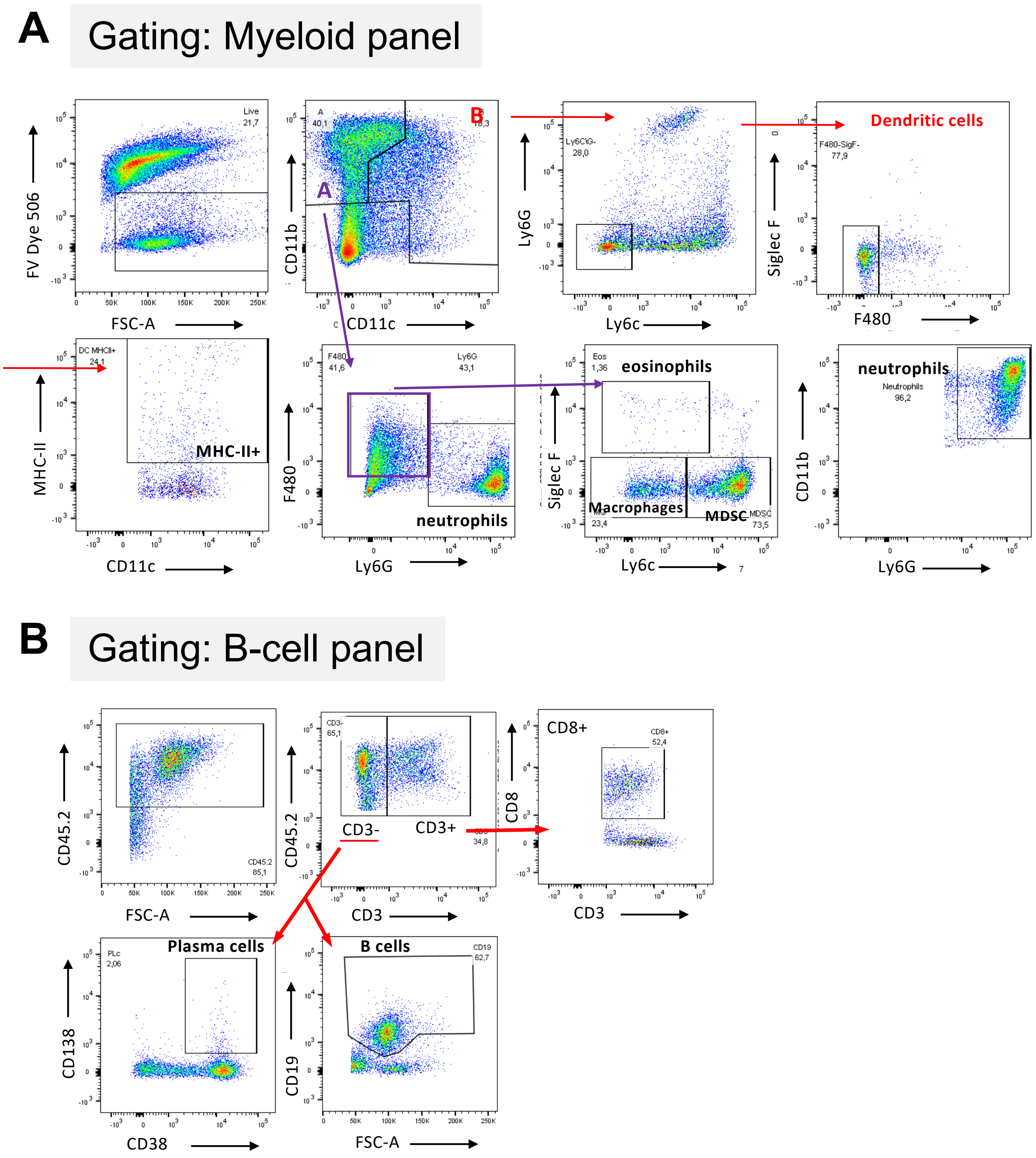

Figure S3. Representative dot plots from CT26LacZ tumour demonstrating the gating strategy used. Leukocytes were first gated based on forward and side-scatter area (FSC-A vs. SSC-A). Doublets were excluded, followed by dead cells, using 7AAD. Subsets were further identified for the (A) Myeloid panel, including neutrophils, eosinophils, myeloid-derived suppressor cells (MDSC) and dendritic cells (DC), by staining of various surface markers. (Neutrophils: CD11b+ F4/80- Ly6G+; Eosinophils: CD11b+ F4/80+ SiglecF+; MDSCs: CD11b+ F4/80+ Ly6C+; DCs:CD11b+ CD11c+). (B) B-cell panel, including CD3+, CD8+, Plasma cells and B-cells. CD8 T cells were identified by positive CD3 and CD8 staining. B cells were identified by negative CD3 staining and positive CD19 staining. Plasma cells were identified by negative CD3 staining, positive CD138 and mid to high CD38 staining.

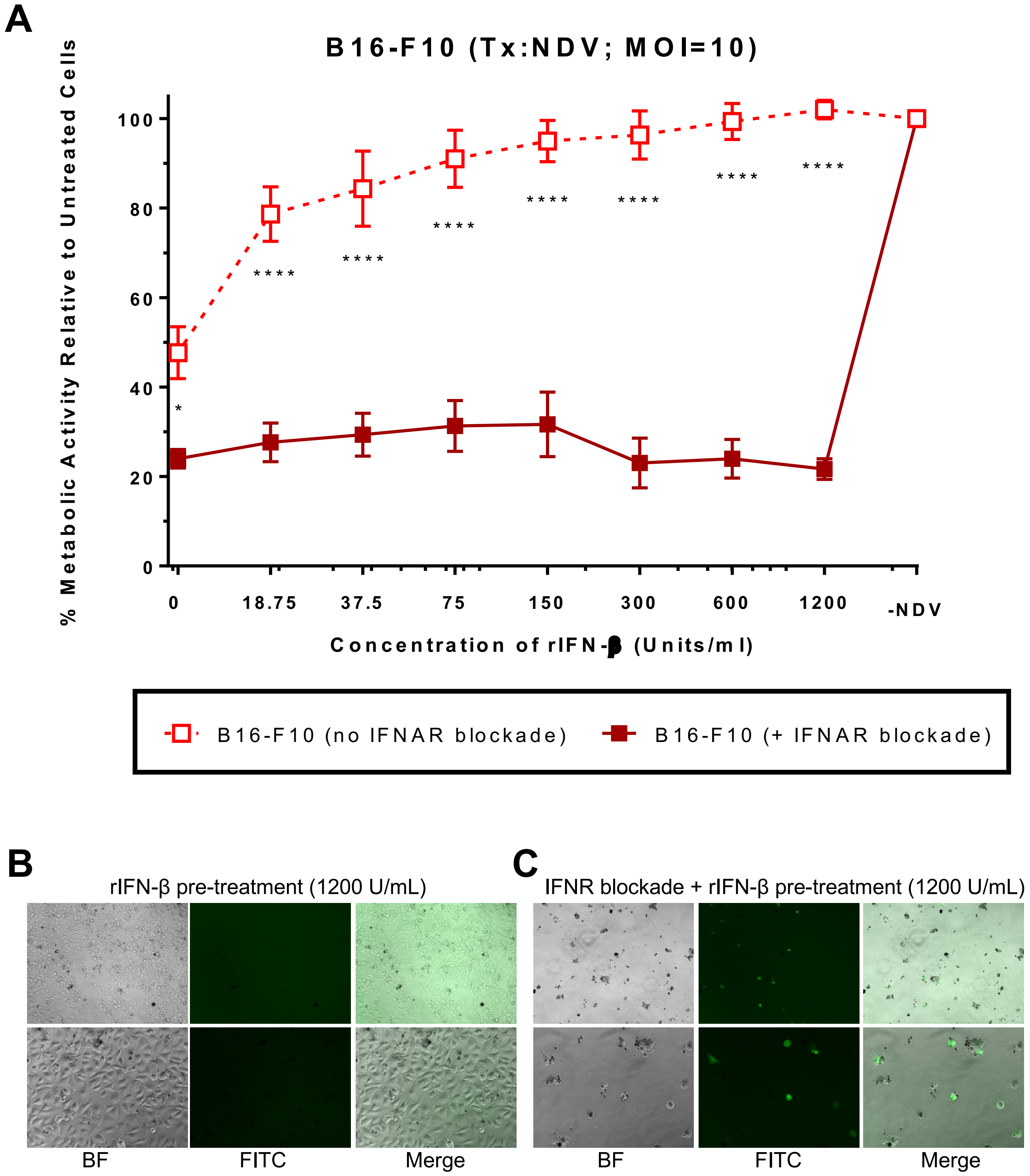

Figure S4. Pre-treatment with interferon (IFN)-β protects murine B16-F10 melanoma cells from NDV-GFP-mediated killing. B16-F10 melanoma cells were pre-treated with or without a type I interferon (IFN) receptor (IFNAR)-blocking antibody for one hour, then IFN-β (0-1200 units/mL) was added for two hours, followed by treatment with NDV-GFP infection at a multiplicity of infection (MOI) of 10. Viability was measured at 72 hours post-infection using a resazurin assay. (A) All graphs show means ± standard error of the mean. Data represents results from three independent experiments with n=3 technical replicates per experiment. Analysis was done using a two-way ANOVA comparing with and without IFNAR blockade results at each time point (*p<0.01, ****<p<0.0001). Representative images of NDV-GFP-infected cells with (B) rIFN-β pre-treatment or with (C) IFNAR blockade + rIFN-β pre-treatment at 48 h post-infection. BF, bright field; FITC, Fluorescein isothiocyanate.

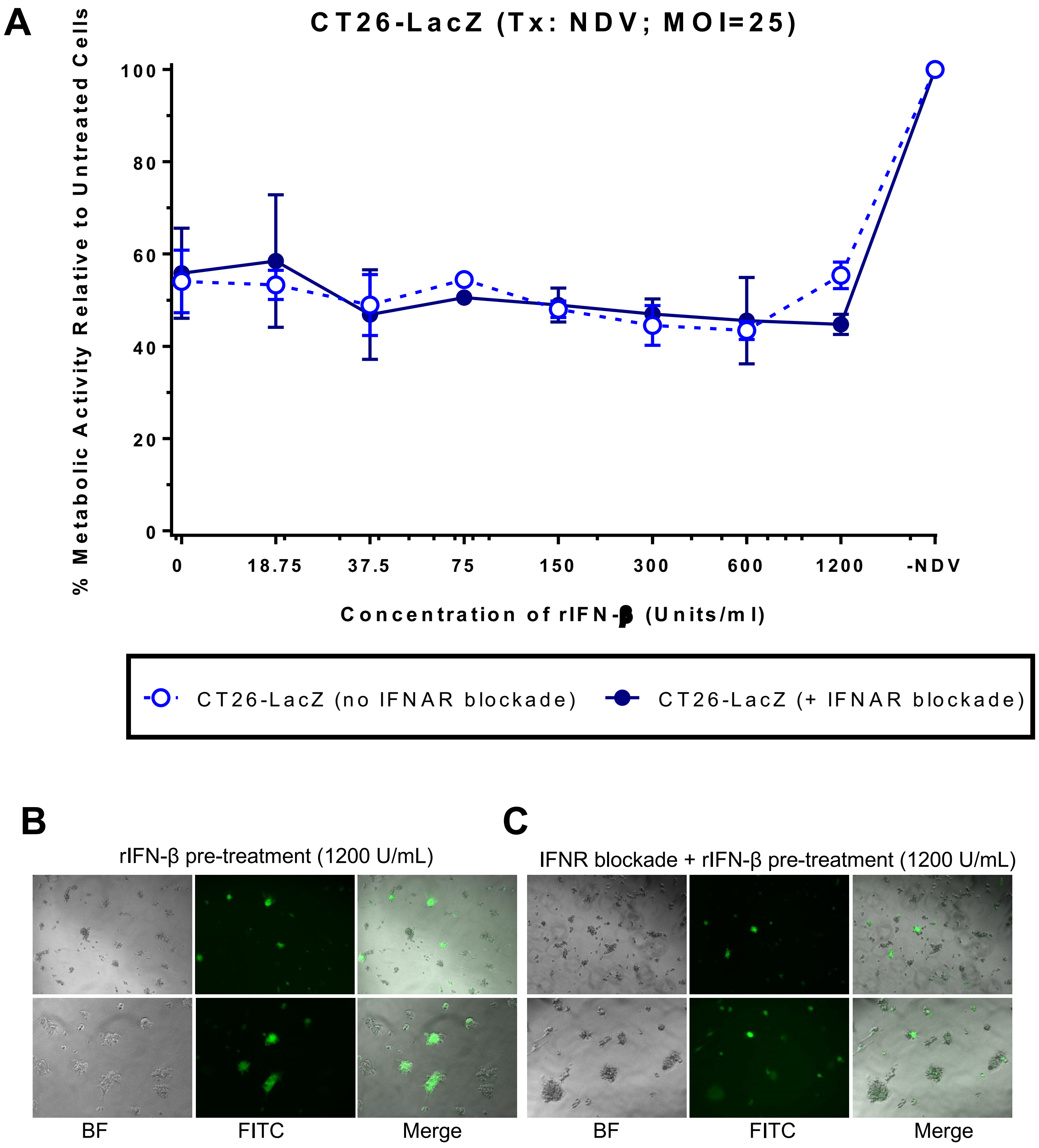

Figure S5. Pre-treatment of murine CT26-LacZ colon carcinoma cells with IFN-β had no effect on NDV(F3aa)-GFP-mediated killing and did not inhibit virus-mediated transgene expression. CT26-LacZ cells were pre-treated with or without a type I interferon (IFN) receptor (IFNAR)-blocking antibody for one hour, then IFN-β (0-1200 units/mL) was added for 2 hours, followed by treatment NDV(F3aa)-GFP infection at a multiplicity of infection (MOI) of 10. Viability was measured at 72 hours post infection using a resazurin assay. (A) All graphs show means ± standard error of the mean. Data represents results from three independent experiments with n=3 technical replicates per experiment. Analysis was done using a two-way ANOVA comparing with and without IFNAR blockade results at each time point (*p<0.01, ****<p<0.0001). Representative images of NDV(F3aa)-GFP-infected cells with (B) rIFN-β pre-treatment or with (C) IFNAR blockade + rIFN-β pre-treatment at 48 hours post-infection. BF, bright field; FITC, Fluorescein isothiocyanate.

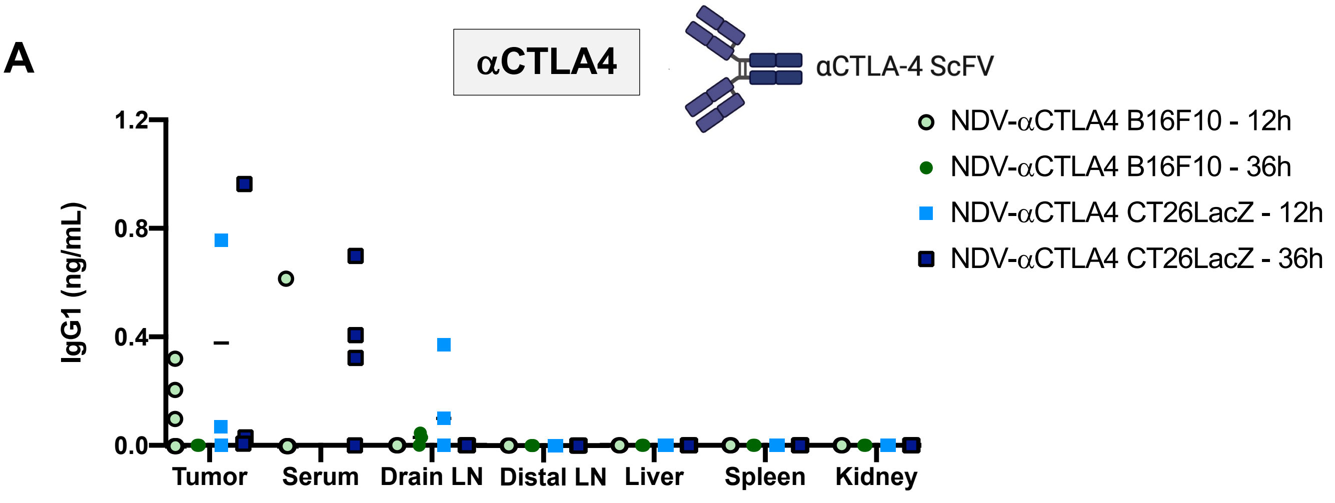

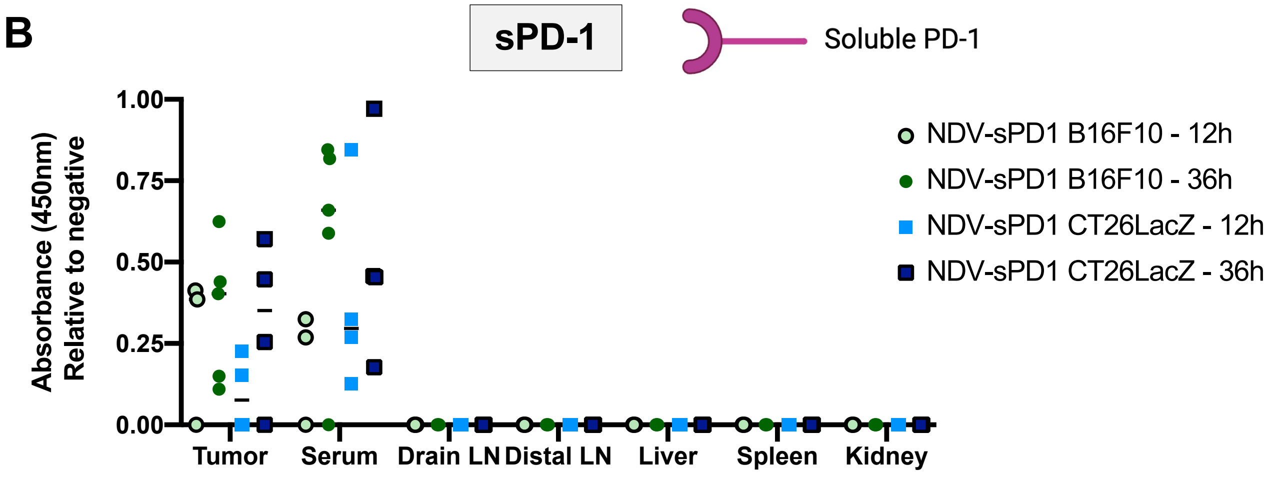

Figure S6. Biodistribution of NDV-expressed immune checkpoint inhibitors (ICIs) following intratumoral administration. Approximately ten-week-old female mice (n=4) bearing B16-F10 (intradermal) or CT26LacZ (subcutaneous) tumors were intratumorally administered 5x10^{7} plaque forming units (PFU) of NDV-aCTLA-4 (Cytotoxic T-lymphocyte-associated protein 4) or NDV-sPD-1 (soluble programmed cell death protein 1). Mice were euthanized either 12 or 36 hours post-NDV injection and blood, tumor, tumor-draining lymph nodes (LN), distal LNs, livers, spleens, and kidneys were harvested. Clarified tissues homogenates and serum samples were diluted 1:10 and a-CTLA-4 concentrations were determined using a commercial human IgG quantitative ELISA and graphed as IgG in ng/mL (A). sPD-1 was detected using an indirect ELISA and OD_{450nm} values were graphed (B). Horizontal lines "-" on the graph indicate means.

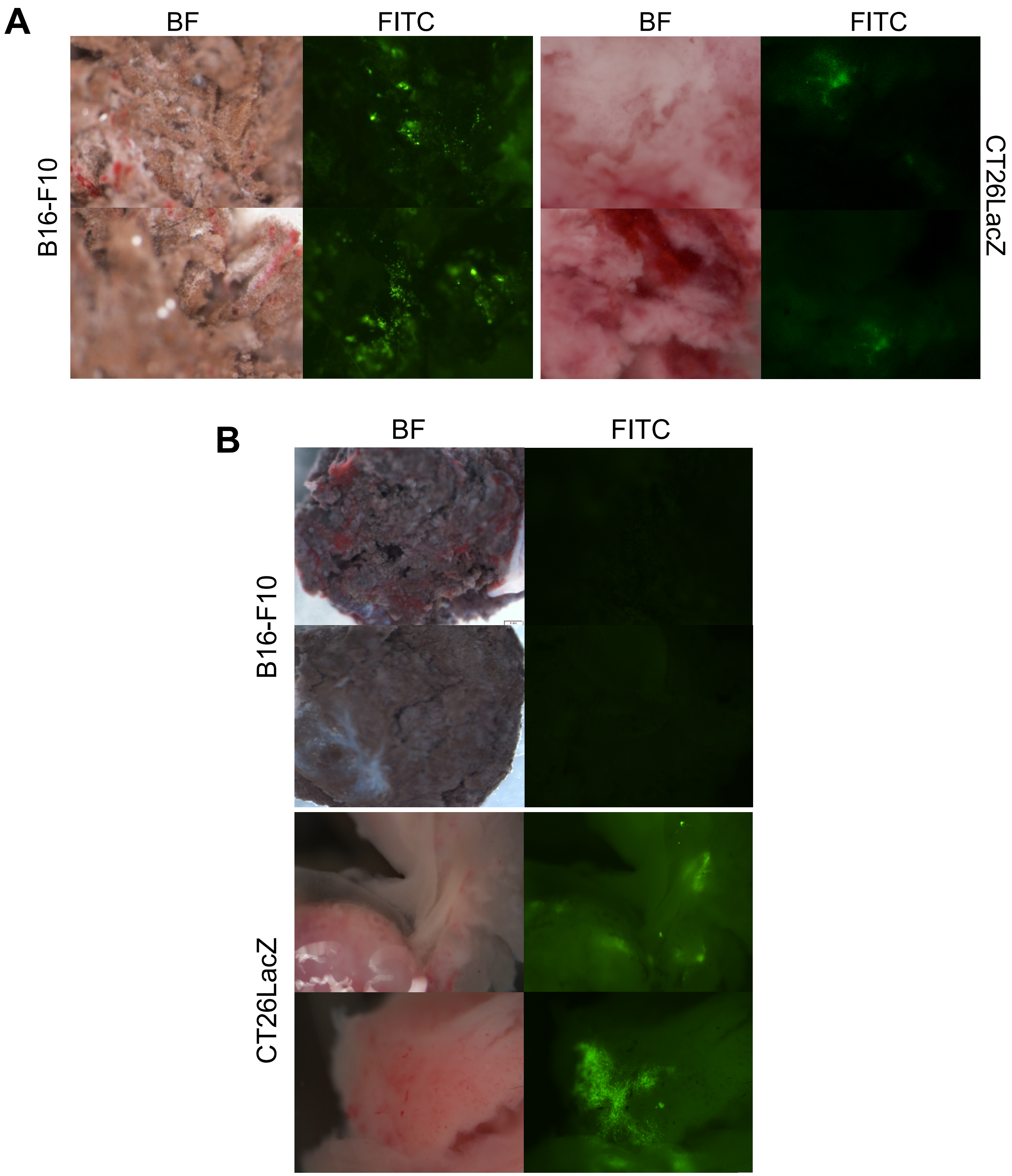

Figure S7. *Ex vivo* imaging of NDV-mediated GFP expression in tumors. (A) Representative images of NDV-mediated GFP expression in murine B16-F10 melanoma and CT26LacZ colon carcinoma tumors injected with $5x10^7$ PFU of NDV-GFP and imaged 12 hours later. (B) Representative images of NDV-mediated GFP expression in B16-F10 and CT26LacZ tumors 24 hours after the third injection of $5x10^7$ PFU every other day. Images were taken using a stereo microscope. Left: Bright-field (BF), Right: FITC (Fluorescein isothiocyanate).

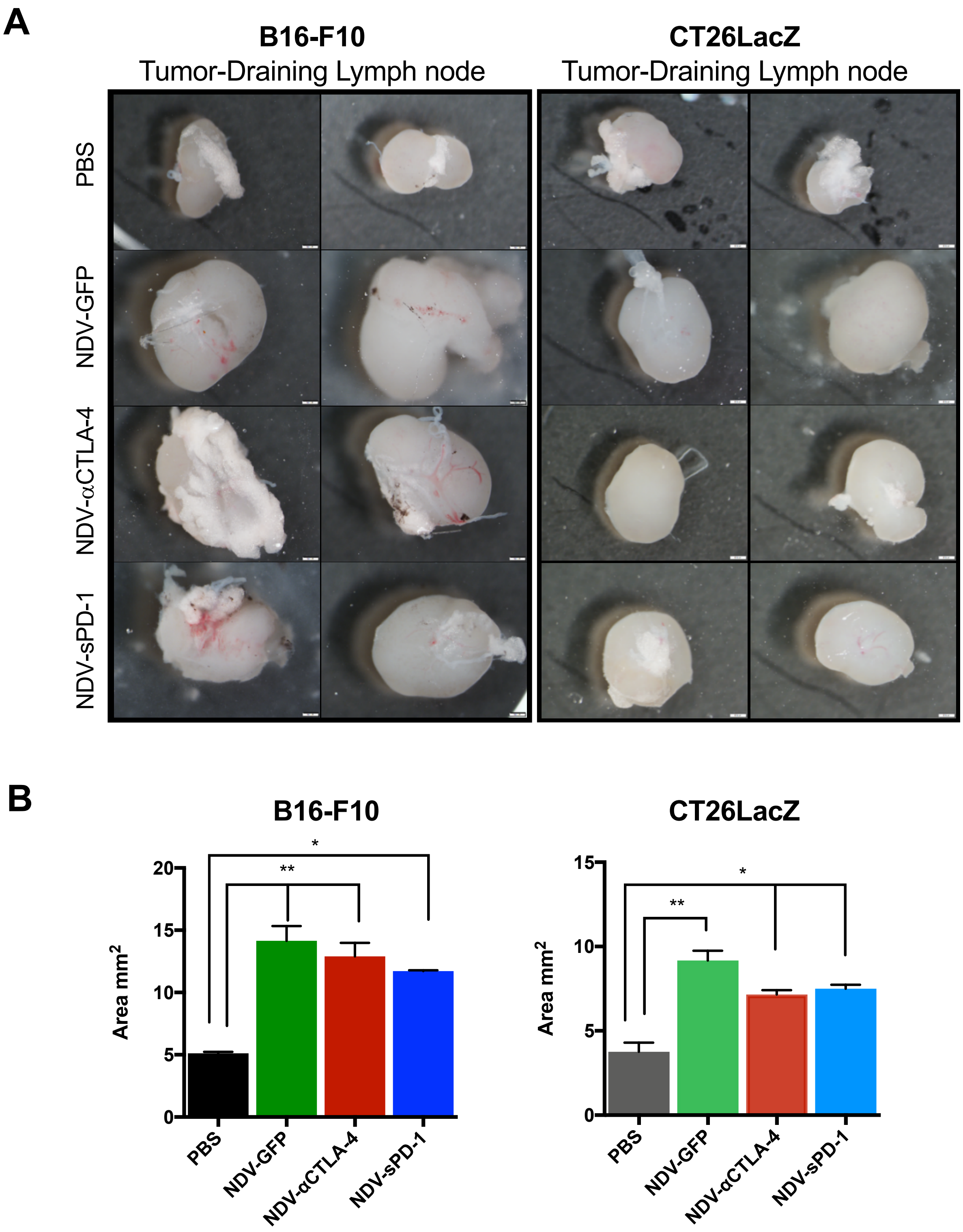

Figure S8. Treatment with rNDVs increased the size of tumor-draining lymph nodes. (A) Two representative images per group of the draining inguinal lymph nodes (LNs) from C57BL/6 B16-F10 and Balb/c CT26LacZ tumor-bearing mice showing the notable increase in size of the rNDV-GFP, rNDV-αCTLA-4, and NDV-sPD-1-treated mice in comparison to PBS-treated mice 36 hours after the third intratumoral injection of $5x10^7$ plaque forming units (PFU) every other day. (B) The area (mm^2) was calculated using ImageJ and analyzed using a one-way ANOVA with Tukey's multiple comparison test; *$p<0.05$, **$p<0.01$.

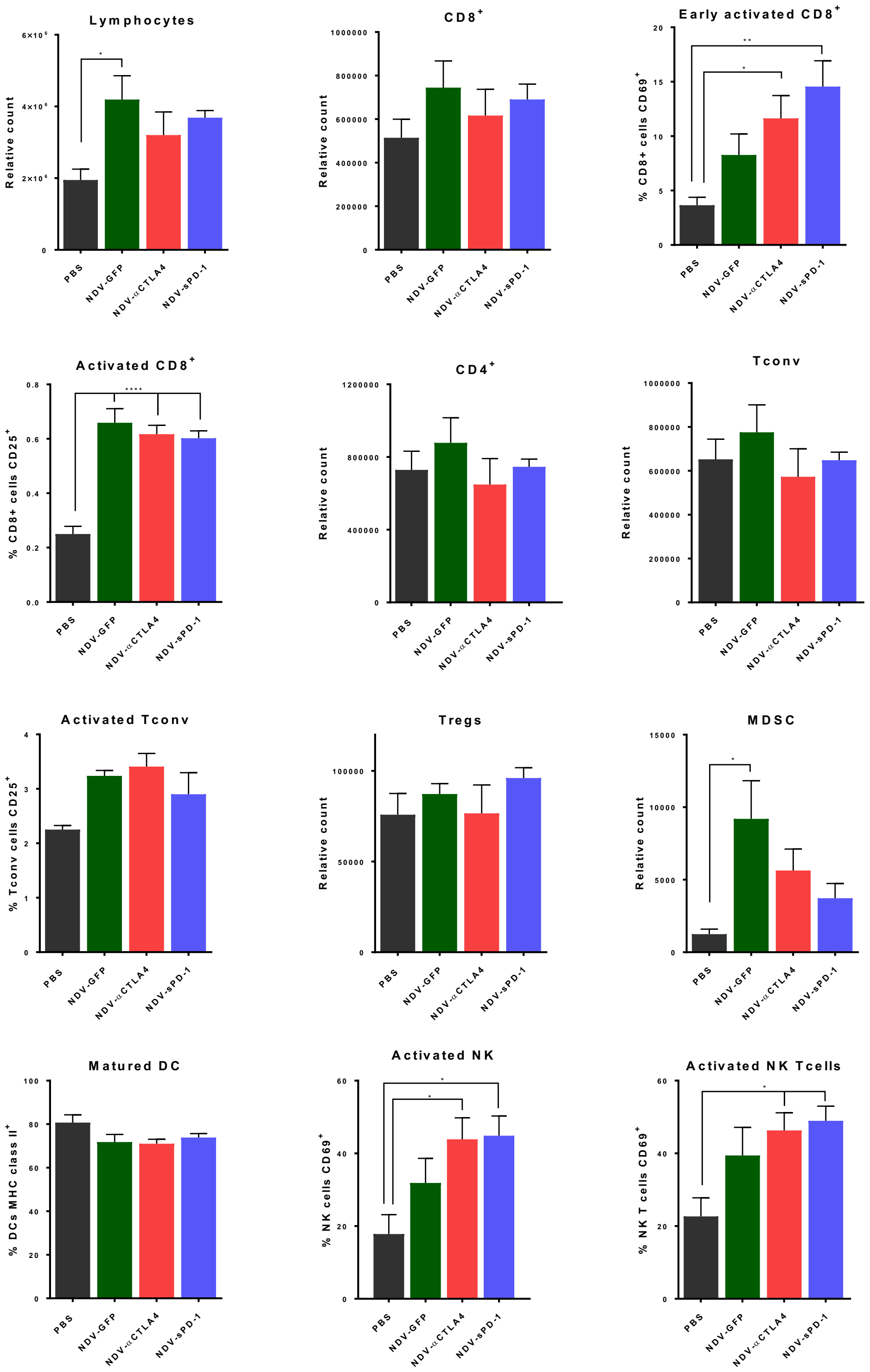

Figure S9. Relative numbers of leukocyte subsets from inguinal lymph nodes (LN) of female B16-F10-tumor bearing C57BL/6 mice 36 hours after recombinant Newcastle disease virus (NDV) expressing green fluorescent protein (GFP), anti-cytotoxic T lymphocyte antigen-4 (αCTLA-4), or secretory programmed cell death protein-1 (sPD-1), every other day for a total of three doses. Numbers were calculated and expressed as relative number or frequencies of cells per LN. Activated conventional (Tconv) $CD4^+$ and $CD8^+$ T cells were expressed as the percentage of these subsets that expressed the activation marker CD25 or CD69, respectively. All graphs show means + standard errors. Data represent results from two independent experiments with n=4-5 mice per group analyzed by one-way analysis of variance (ANOVA). *$P<0.05$, **$P<0.01$, ***$P<0.001$, ****$P<0.0001$. MDSC (Myeloid-derived suppressor cells), Tconv (conventional $CD4^+$ T cell), Tregs (regulatory T cell), DC (dendritic cells), and NK (Natural Killer cell).

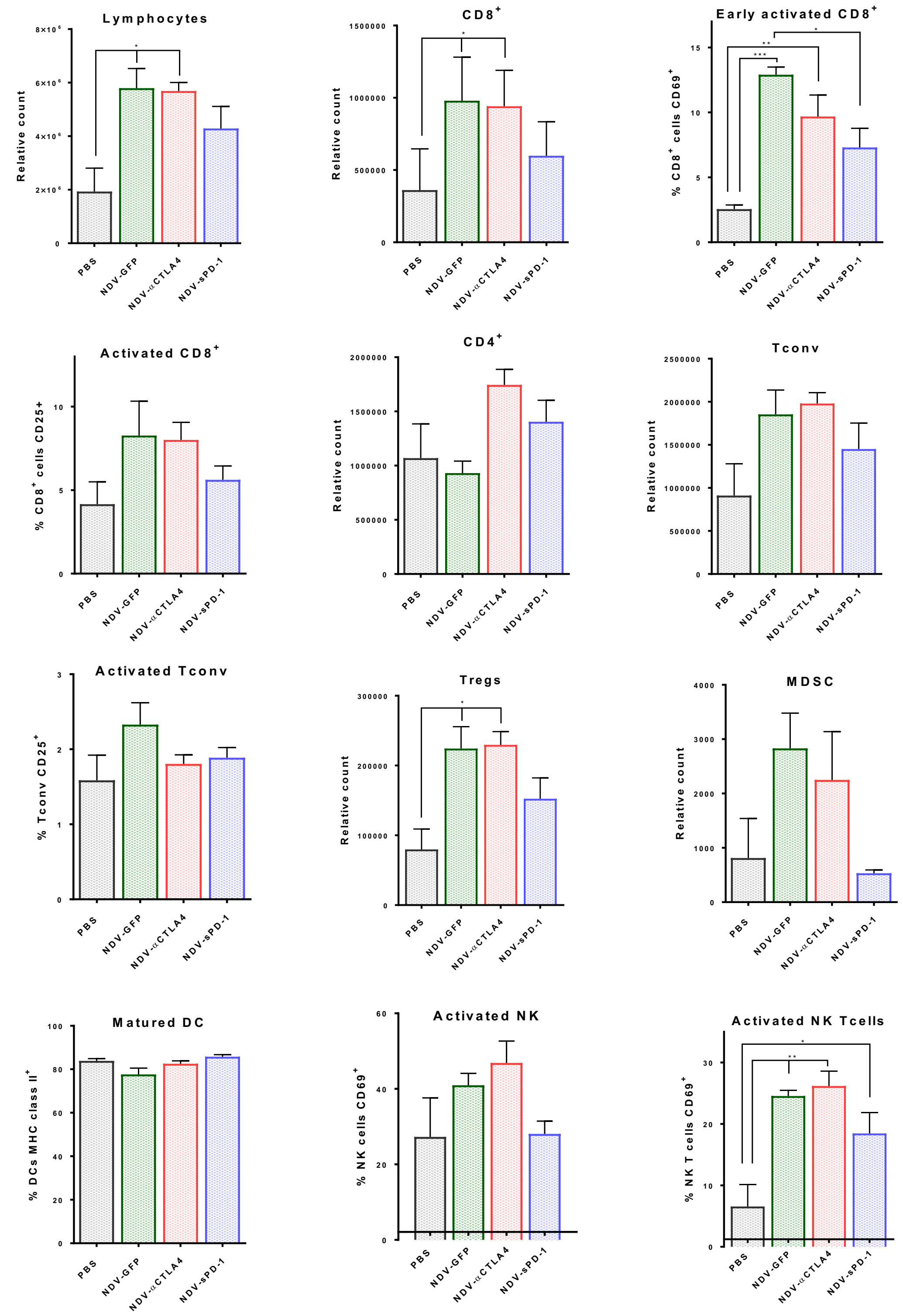

Figure S10. Relative numbers of leukocyte subsets from inguinal lymph nodes (LN) of female CT26LacZ-tumor bearing Balb/c mice 36 hours after recombinant Newcastle disease virus (NDV) expressing green fluorescent protein (GFP), anti-cytotoxic T lymphocyte antigen-4 (αCTLA-4), or secretory programmed cell death protein-1 (sPD-1), every other day for a total of three doses. Numbers were calculated and expressed as relative number or frequencies of cells per LN. Activated conventional (Tconv) $CD4^+$ and $CD8^+$ T cells were expressed as the percentage of these subsets that expressed the activation marker CD25 or CD69, respectively. All graphs show means + standard errors. Data represent results from two independent experiments with n=4-5 mice per group analyzed by one-way analysis of variance (ANOVA). *P<0.05, **P<0.01, ***P<0.001, ****P<0.0001. MDSC (Myeloid-derived suppressor cells), Tconv (conventional $CD4^+$ T cell), Tregs (regulatory T cell), DC (dendritic cells), and NK (Natural Killer cell).
